# A Health Media Literacy Intervention Increases Skepticism of Both Inaccurate and Accurate Cancer News Among U.S. Adults

**DOI:** 10.1093/abm/kaae054

**Published:** 2024-09-26

**Authors:** Benjamin Lyons, Andy J King, Kimberly A Kaphingst

**Affiliations:** Department of Communication, University of Utah, Salt Lake City, USA; Cancer Control and Population Sciences, Huntsman Cancer Institute, Salt Lake City, USA; Department of Communication, University of Utah, Salt Lake City, USA; Cancer Control and Population Sciences, Huntsman Cancer Institute, Salt Lake City, USA; Department of Communication, University of Utah, Salt Lake City, USA; Cancer Control and Population Sciences, Huntsman Cancer Institute, Salt Lake City, USA

**Keywords:** Media literacy, Cancer, Misinformation, Health communication

## Abstract

**Background:**

Inaccurate cancer news can have adverse effects on patients and families. One potential way to minimize this is through media literacy training—ideally, training tailored specifically to the evaluation of health-related media coverage.

**Purpose:**

We test whether an abbreviated health-focused media literacy intervention improves accuracy discernment or sharing discernment for cancer news headlines and also examine how these outcomes compare to the effects of a generic media literacy intervention.

**Methods:**

We employ a survey experiment conducted using a nationally representative sample of Americans (*N* = 1,200). Respondents were assigned to either a health-focused media literacy intervention, a previously tested generic media literacy intervention, or the control. They were also randomly assigned to rate either perceived accuracy of headlines or sharing intentions. Intervention effects on accurate and inaccurate headline ratings were tested using OLS regressions at the item-response level, with standard errors clustered on the respondent and with headline fixed effects.

**Results:**

We find that the health-focused media literacy intervention increased skepticism of both inaccurate (a 5.6% decrease in endorsement, 95% CI [0.1%, 10.7%]) and accurate (a 7.6% decrease, 95% CI [2.4%, 12.8%]) news headlines, and accordingly did not improve discernment between the two. The health-focused media literacy intervention also did not significantly improve sharing discernment. Meanwhile, the generic media literacy intervention had little effect on perceived accuracy outcomes, but did significantly improve sharing discernment.

**Conclusions:**

These results suggest further intervention development and refinement are needed before scaling up similarly targeted health information literacy tools, particularly focusing on building trust in legitimate sources and accurate content.

## Teaser

Adults who participated in a health media literacy intervention became more skeptical of both accurate and inaccurate cancer news, but showed no improvement in distinguishing between the two.

## Introduction

Researchers have been concerned about health misinformation for decades, and the COVID-19 pandemic brought calls for systematic study of the accuracy and quality of health information—particularly online [1]. As a result, there is significant evidence that cancer-related misinformation, especially regarding alternative therapies and supposed miracle cures, is extensive [2–4]. Indeed, other research suggests that exposure to—and belief in—cancer treatment misinformation on social media is widespread, at least in the United States [5]. This is important because, although rare, alternative treatment use for curable cancer is associated with a greater risk of death [6]. While explicit concern for misinformation has grown in the past few years, there are parallel concerns that people may reject reliable, accurate sources of information [7]. Because accurate cancer knowledge is linked to positive behavioral outcomes [8] and cancer misperceptions may impede healthcare decision-making and may exacerbate existing health inequalities [9], it is imperative to improve public discernment of accurate and inaccurate cancer information in online news.

Although research describing health misinformation in the public communication environment flourishes across topics, few studies attempt to take a proactive approach toward improving health media literacy (and, as a result, discernment of health information). However, researchers have suggested improving health literacy is likely a key to mitigating the effects of health misinformation [10]. Although mitigation strategies like debunking offer promise for addressing specific myths and mistruths, skills-based interventions are much more scalable, and their guidelines can be applied to new (mis)information as it is encountered. For example, research has found that general digital literacy interventions can improve discernment of accurate and inaccurate news content [11, 12]. These studies, though, have focused on political content, or politicized health topics like COVID-19, and no studies to our knowledge have tested the effects of similar literacy interventions on health domain-specific content (particularly about chronic illnesses like cancer).

As such, the current study draws on a representative sample of American adults to test whether a health-focused media literacy intervention can improve discernment between accurate and inaccurate cancer news in the United States. In addition to a control condition, the health-focused media literacy intervention is tested against an existing generic media literacy intervention, which has demonstrated general effectiveness previously, as a baseline comparison group. Using this design, we ask whether the health-focused media literacy intervention, developed but not previously tested at a population scale, improves recognition of inaccurate content and if it does so beyond the baseline. In addition, we ask whether this intervention has any unintended spillover effects on accurate news evaluations. Finally, we explore whether the intervention is more or less successful among those predisposed to endorse health-related misinformation.

### Media Literacy Tips

**Fig. 1.**
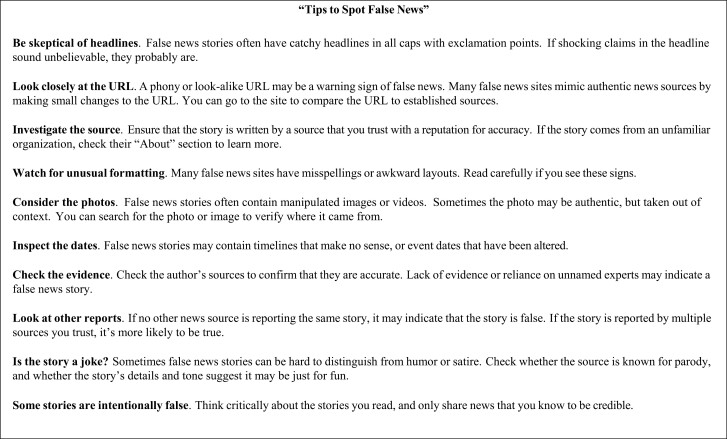
Generic media literacy intervention (News Tips).

Brief media literacy tips [11], such as those developed and promoted by Facebook to help users spot false news (see Fig. 1), have been shown to be effective in improving accuracy discernment in other topical news domains (i.e., political news), both in the U.S. and in numerous other countries [11, 12]. Further, these improvements appear to endure for several weeks, suggesting their effects are not ephemeral—such as priming a concern for accuracy—but instead reflect some degree of learning.

Importantly, tips-based interventions require minimal time or effort to apply, in contrast to long-term media literacy programs that typically aim to increase critical thinking in the long term at the expense of speed and scale [13]. Tips-style interventions offer concise guidance presented as practical actions [11]. These sorts of literacy tips typically prompt individuals to approach online media content with greater mindfulness and skepticism [14, 15].

### Focusing on Health News

Although generic media literacy interventions might be generally useful in guiding news evaluation, health news—and health misinformation—has its own unique properties [16]. Health news tends to emanate from institutions and experts, but their credibility can be hijacked, and expertise can be misrepresented [17]. There are also thriving online communities of wellness and other influencers who compete with legitimate experts for attention [18, 19]. These influencers often share unsupported claims on social platforms in order to monetize antiexpert positions [20]. Aside from source differences, health news differs from public affairs and political coverage in terms of content. For instance, health news often features inherently uncertain scientific findings that can be easily misinterpreted or misapplied (e.g., reporting on lab advances in cancer etiology as a potential source of cure) [21, 22]. Lastly, health news is often directly relevant to the potential health outcomes of readers or their families, so readers might be especially susceptible to misleading news promising better outcomes [23].

Given the unique nature of health and medical news, media literacy interventions need to be tested beyond previous applications focusing on politics and public affairs because their effects may be domain-sensitive in unpredictable ways. Further, the development of new, health-focused media literacy tips could allow for more specific applications for readers and prepare them for signs of bias exclusive to the health news domain. Our plan to test a previously supported intervention specific to public affairs and politics, alongside a health-focused media literacy intervention, advances research in this area through comparison and innovation.

### Considering Unintended Intervention Effects

There are also some potential negative unintentional effects of these interventions. In prior work, generic media literacy tips were shown to reduce the perceived accuracy of not only false news headlines but also, to some extent, mainstream news headlines [11]. Although this resulted in better discernment overall and better discernment based on simulations using existing news consumption patterns, this finding is consistent with an expanding body of research indicating that certain information integrity interventions, despite their upsides, might inadvertently lead to adverse effects on the evaluation of authentic information [14, 24].

Given the variety of effects different interventions appear to have on the perceived accuracy of accurate and inaccurate news [13], unintended spillover effects may be sensitive to small changes in tip sets. They also may be domain specific. Therefore, it is imperative to test for these when examining a new intervention’s efficacy.

### Differential Efficacy Across Populations

Intervention effects vary across segments of the population. Some groups have been shown to be especially vulnerable to misinformation in general and about science and medical topics in particular. It is especially important to consider how interventions work among these individuals because they have a higher propensity to fall for inaccurate claims. Specifically, antiexpert sentiments, conspiracism, and disaffection for the media tend to be associated with endorsement of unsupported beliefs more broadly, while unfavorable views toward domain-specific experts and authorities (e.g., pharmaceutical companies, doctors, and scientists) are often associated with health misperceptions [25]. At the most domain-specific level, it is important to consider individuals holding more preexisting misperceptions about cancer (e.g., inaccurate beliefs about risk factors like stress, 5G technology exposure, or GMO consumption) because new inaccurate claims may fit with their prior beliefs. By way of confirmation bias and fluency effects, these new claims may be more likely to be accepted, while accurate claims may be rejected because they do not comport with those prior beliefs [26, 27]. Ideally, an effective intervention would not only work equally well among respondents holding these views as with the broader public but would be more effective among them, given that they have a greater propensity to hold misperceptions in the first place.

### Hypotheses and Research Questions

Based on the above, we pose a series of preregistered hypotheses and research questions (https://osf.io/984t5/?view_only=b4453e6cc1d643cfa3562a20fd9e648c). Before examining intervention effects, we first assess observational outcomes for existing digital literacy and health literacy scales to determine if these correlate with perceived accuracy/sharing intent for inaccurate cancer news in our control condition. We also explore other potential correlates of these outcomes.

RQ0: Do digital literacy or health literacy correlate with inaccurate cancer news perceived accuracy, sharing intent, or discernment? What are other correlates of these outcomes?

Then, we move to intervention outcomes. We hypothesize that the generic media literacy intervention (a) reduces perceived accuracy/sharing intent for inaccurate headlines and (b) improves discernment scores compared to control. We expect that the health-focused media literacy intervention will perform similarly.

H1. A generic media literacy intervention reduces perceived accuracy/sharing intent for inaccurate headlines and improves discernment.

H2. A health-focused media literacy intervention reduces perceived accuracy/sharing intent for inaccurate headlines and improves discernment.

We then ask if the effects of a health-focused media literacy intervention are larger than those of a generic media literacy intervention.

RQ1. Is the effect of a health-focused media literacy intervention on perceived accuracy/sharing intent or discernment larger than that of a generic media literacy intervention?

Next, we assess potential spillover effects on evaluations of accurate headlines.

RQ2. What are the effects of interventions on perceived accuracy/sharing intent for accurate headlines?

Lastly, we examine whether intervention effects are moderated by pretreatment individual difference measures (cancer risk factor beliefs, antiexpert sentiment, conspiracism, affect toward pharmaceuticals, doctors, scientists, the media, and social media, as well as personal cancer history).

RQ3. Are the effects of interventions moderated by conspiracy ideation, antiexpert views, digital literacy, health literacy, cancer history, trust measures, etc.?

As preregistered, we also assess whether interventions are relatively more effective on perceived accuracy than on sharing intent outcomes in Table D.8.

## Methods

Our data come from a survey experiment (*N* = 1,200) conducted by YouGov from October 25 to November 21, 2023. Respondents were selected by YouGov’s matching and weighting algorithm to approximate the demographic attributes of the U.S. population (see Supplementary Material, Appendix A for a full description of sampling procedures).

Our participants closely resemble the U.S. population in demographics. All descriptive statistics reported here are unweighted. Respondents are 53% female, 45% male, and 2% nonbinary/other. They are 71.8% white, 11.7% Black, 8.8% Hispanic, 2.8% Asian, 0.8% Native American, and 4.8% two or more races or other. Median age is 53 (*M* = 51.6, *SD* = 15.9, range = 18–89), 40% hold a 4-year college degree or higher, 91% had some form of health insurance, and 11.75% reported having a prior cancer diagnosis. Median household income is $50,000–$59,999 (39.9% had an income of $39,999 or less, 33.3% had an income between $40,000 and $79,999, 12.4% had income between $80,000 and $119,999, and 14.1% had an income over $120,000). In Table 1, we report descriptive statistics for all pretreatment variables used in our analyses below.

**Table 1:** Descriptive Statistics of Co-variates and Predictors (*N* = 1200)

Variable	*M*	*SD*	Min	Max
Age	51.61	15.92	18	89
Female	0.53	0.50	0	1
Nonwhite racial background	0.29	0.45	0	1
College degree	0.40	0.49	0	1
Cancer history	0.12	0.32	0	1
“Low-end” digital literacy	1.98	0.71	1	5
Health literacy	4.42	0.71	1	5
Cancer beliefs (true)	3.32	0.69	1	5
Cancer beliefs (false)	2.67	0.73	1	5
Anti-expert sentiment	2.62	0.88	1	5
Conspiracism	3.04	1.02	1	5
Affect toward pharmaceuticals	40.92	26.51	1	100
Affect toward doctors	61.10	26.02	1	98
Affect toward scientists	59.81	27.94	1	99
Affect toward the news media	42.24	28.50	1	98
Affect toward social media	38.91	26.33	1	99

Note: Affect items are measured on 100-pt. scales with higher scores reflecting warmer (favorable) affect.

### Design and Procedures

The study uses a 3 (intervention treatment: a generic media literacy intervention, a health-focused media literacy intervention, control) *×* 2 (outcome measure: perceived accuracy, sharing intent) design. Respondents completed a pre-treatment questionnaire that included measures of all predictors used in our observational analyses, as well as all moderators used in our analyses of treatment effects. They were then randomly assigned to one of the three treatment conditions and also randomly assigned to provide one of the two outcome measures. They then completed a headline evaluation task described below. After evaluating the headlines for accuracy or sharing intent, all participants were informed which of the news headlines they rated were not accurate and not linked to current scientific knowledge. This debrief also provided links to accurate cancer information if participants were interested, though we did not track if participants clicked out during the debrief.

### Generic Media Literacy Intervention (News Tips)

Our selected generic media literacy intervention (referred to as News Tips below) has been tested in several other studies [11, 12] and replicates Facebook’s “Tips to Spot False News,” which were developed in collaboration with the nonprofit First Draft and subsequently promoted at the top of users’ News Feeds in 14 countries in April 2017 and printed in full-page newspaper ads in the USA, UK, France, Germany, Mexico, and India [28–33]. This intervention provides simple rules that can help individuals evaluate the credibility of sources and identify indicators of problematic content without expending significant time or attention. For instance, one sample tip recommends that respondents “[b]e skeptical of headlines,” warning that “[i]f shocking claims in the headline sound unbelievable, they probably are.”

### Health-Focused Media Literacy Intervention (BOAST)

Our health-focused media literacy intervention, meanwhile, is based on a set of tips developed by the nonprofit organization Facing Our Risk of Cancer Empowered (FORCE) called BOAST. Note that the BOAST intervention, as presented on the FORCE website, extends beyond the brief tips we test, as it provides an interactive intervention that leads users through modules about each BOAST tip category (i.e., biased, overblown, amateur, sales-focused, and taken out of context/too early to be useful). While also providing simple rules that can be applied without expending significant time or attention, these tips more directly target misleading health claims (https://www.facingourrisk.org/BOAST/). For instance, one rule called “Too early to be useful” warns that “[s]ometimes articles cover research without directly saying it’s too early to be useful. It can take a long time for studies and research to become actionable advice about a person’s health.” Another rule, “Amateur,” warns that “[w]hen it comes to health information, it’s important to rely on experts, not amateurs.” As such, the guidelines advocated in this intervention are more specific to health media content. We test an abbreviated tips-style version of BOAST as this enhances its scalability and is more readily compared to other brief media literacy tips interventions, which have been shown to be broadly effective. Although it is possible this abbreviation could impact our findings, we would expect this to lead to more conservative estimates.

**Fig. 2.**
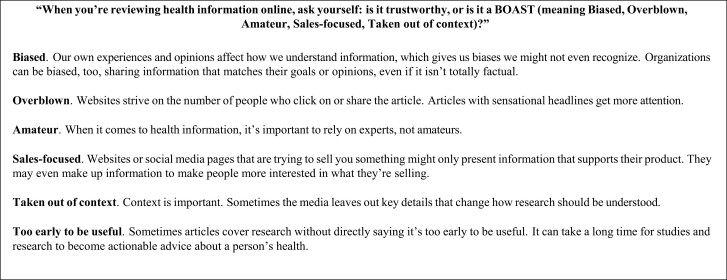
Health-focused media literacy intervention (BOAST).

The News Tips and BOAST intervention text is shown in Figs. 1 and 2, respectively. Each intervention’s tips were broken into three sets when displayed to respondents. Respondents were held on each set’s page for four seconds before they could progress. After each set, respondents answered a comprehension check question asking them to identify one of the tips they had just read. In the generic tips condition, 64% of respondents got all three checks correct; 87% got at least two correct (*M* = 2.49, *SD* = 0.76). In the BOAST condition, 49% of respondents got all three correct, 84% got at least two correct (*M* = 2.28, *SD* = 0.84). We include balance checks in Tables D.1 and D.2.

As mentioned, prior to the headline evaluation task, respondents were randomized into two further groups: to evaluate the *accuracy* of headlines or to evaluate their *sharing intentions* of headlines. We split respondents into these two response conditions because we are interested in the effects on both outcomes, but prior work suggests asking the outcome items sequentially can bias responses [34].

### Cancer News Evaluation Outcomes

We exposed respondents to 18 article headlines relating to cancer and had those respondents evaluate each headline. The articles, all of which were actual online news stories available to us prior to our field date, were published by actual online sources. Respondents rated the accuracy, or their sharing intention, for 6 inaccurate headlines (randomly drawn from a pool of 16) and 12 accurate headlines (randomly drawn from a pool of 44). We presented respondents with a 2:1 accurate/inaccurate ratio (rather than 1:1) to better mirror the prevalence of dubious content in the actual information environment [11]. Headlines are presented in Supplementary Material, Appendix B. We randomized headline order for all respondents. Headlines were presented as they would appear on a Facebook News Feed to enhance ecological validity (featuring a headline, photo, and the news source’s web domain).

Perceived accuracy for each item was measured on a 4-pt. scale (“Not at all accurate” (1) to “Very accurate” (4)). Sharing intent was likewise measured on a 4-pt. scale (“Not at all likely” (1) “Very likely” (4)). In the accuracy-outcome condition, we looked at perceived accuracy for each news type (accurate and inaccurate) at the headline (item) level. We also examined ability to distinguish accurate from inaccurate cancer news—*discernment*—as the mean for accurate news accuracy *minus* the mean for inaccurate news accuracy at the respondent level. In the sharing outcome condition, we looked at sharing intention for each news type (accurate and inaccurate) at the headline (item) level. We also examined *sharing discernment* as the mean of accurate news sharing intent *minus* the mean of inaccurate news sharing intent at the respondent level.

### Demographics, Correlates, and Moderators

We collected additional pretreatment variables for use as covariates and predictors in observational analyses, and as moderators in tests of heterogeneous treatment effects. Descriptive statistics for each variable are listed in Table 1. Full question wording is included in Supplementary Material, Appendix A. Regarding scaled items, we employed a “low-end” digital literacy scale developed to separate low-skill users from the rest of the population [35], where high scores indicate more self-reported difficulty with tasks relating to online information (four items, Cronbach’s alpha = .64), and the Brief Health Literacy Screen (BHLS), standard measure of subjective health literacy [36] (four items, Cronbach’s alpha =.77). Further, we used previously validated scales for antiexpert sentiment (three items, Cronbach’s alpha = .75) and conspiracism (four items, Cronbach’s alpha = .87) [37].

### Analytic Approach

#### Observational analyses

We conducted all analyses using Stata 18. For RQ0, we first employed zero order correlations of digital literacy and health literacy with our outcome measures: perceived accuracy of (i) inaccurate and (ii) accurate cancer news headlines, and (iii) accuracy discernment, as well as intentions to share (iv) inaccurate and (v) accurate cancer news, and (vi) sharing discernment. These tests are conducted using the control group, who saw no media literacy intervention treatment. Next, we conducted a series of OLS regressions with these same outcome measures. For each outcome, we first included digital literacy and health literacy as predictors. To assess the robustness of any of these potential relationships, and to determine other potential correlates of cancer news discernment, this was followed by a model that also included a series of additional potential predictors (cancer beliefs [true], cancer beliefs [false], antiexpert sentiment, conspiracism [i.e., disposition to believe in conspiracies], and affect toward pharmaceuticals, doctors, scientists, the media, and social media) [25, 26]. All models included a set of standard demographic covariates (age, sex, education, nonwhite, and cancer history).

Models of perceived accuracy of headlines and intent to share headlines are conducted at the headline (item) level, rather than averaging across all inaccurate or accurate items, as an averaging approach “obscures potential variation that exists across items” [38]. We included fixed effects for each item to account for differing baseline levels of plausibility and cluster on respondent to account for correlations between their ratings across items. Because they are computed as difference scores, discernment outcomes are modeled at the respondent level.

### Intervention Analyses

To test the main effects of the interventions across outcomes (H1, H2, RQ1, and RQ2), we used a series of OLS regression models with robust standard errors. We employed separate models for accurate and inaccurate headlines, as well as a model of discernment between these. Models of perceived accuracy of headlines and intent to share headlines are conducted at the headline (item) level, and we included fixed effects for each item to account for differing baseline levels of plausibility and cluster on respondent to account for correlations between their ratings across items [38]. Discernment outcomes are modelled at the respondent level. After these were conducted, we computed point estimates for the linear combination of the treatments’ OLS regression coefficients (i.e., BOAST *minus* News Tips) and tested for differences between them.

To test for differential intervention effects (across cancer beliefs [true], cancer beliefs [false], antiexpert sentiment, conspiracism, and affect toward pharmaceuticals, doctors, scientists, the media, and social media, as well as personal cancer history) (RQ3), our modeling choices mirrored our tests of main effects, with the addition of each predictor and its interactions terms for each treatment (i.e., we tested each predictor separately rather than in an omnibus model).

### Smallest Detectable Effect Sizes

We calculated some estimates for smallest detectable effect sizes (SDES), given our models. For item-level regression analyses of accurate items, we have the parameters of *N* = 600 respondents (in either the accuracy or sharing groups), 12 repeated measures per respondent with an intraclass correlation (ICC) of about 0.3, and two predictors (the treatment arms). Assuming a significance level of 0.05 and power of 0.80, the SDES is about *d* = 0.07. For item-level analyses of inaccurate items, for which we have 6 repeated measures and an ICC of about 0.4, the SDES is about *d* = 0.08. For exploratory moderation tests, which include an additional independent variable and interaction terms with each treatment, these estimates remain *d* = 0.07 and *d* = 0.08, respectively (while the inclusion of interaction terms increases the number of predictors and reduces the degrees of freedom, the effect is minimal at this sample size). For our respondent-level regression analyses of discernment, we have a simpler set of parameters: *N* = 600 respondents, and two predictors (treatment arms). In this case, the SDES is about *d* = 0.12. When considering exploratory moderation tests, which each include an additional independent variable and two interaction terms, the estimate remains about *d* = 0.12.

## Results

### Observational Results: Correlates of Perceived Accuracy and Sharing Intent

First, we note that on average, respondents rated the accuracy of inaccurate headlines lower (*M* = 1.96, *SD* = 0.65) than that of accurate headlines (*M* = 2.61, *SD* = 0.49) in the control condition, and average sharing intent for inaccurate headlines (*M* = 1.76, *SD* = 0.84) was lower than that for accurate ones (*M* = 1.90, *SD* = 0.84) among this group as well (note also that sharing intent is low regardless of veracity). Histograms for these outcomes are shown in Fig. D.1.

With this in mind, we assessed whether existing digital literacy or health literacy self-report scales correlated with the perceived accuracy of (in)accurate news, intent to share it, accuracy discernment, or sharing discernment. These tests are conducted using the control group, who saw no media literacy intervention treatment. We first tested for zero-order correlations with each of our correlates as shown in Table 2. These show that low-end digital literacy (i.e., self-reported struggles with basic digital tasks) is moderately correlated with greater perceived accuracy of inaccurate headlines (*r* = .24, *p* < .001), lower accuracy discernment (*r* = −.19, *p* = .008), and greater sharing intent for inaccurate headlines (*r* = .23, *p* = .001). Health literacy was less strongly associated with these outcomes. Both scales showed weak associations with sharing discernment.

**Table 2:** Zero-Order Correlation Coefficients for Literacy Scales and News Discernment Outcomes in the Control Condition

	Low-end digital literacy	Health literacy
Inaccurate headline perceived accuracy	0.24***	−0.16*
Accuracy discernment	−0.19**	0.14*
Inaccurate headline sharing intent	0.23***	−0.16*
Sharing discernment	−0.07	0.05

Note: * *p* < .05, ** *p* < .01, *** *p* < .005.

Next, we conducted a series of OLS regressions with our outcome measures: perceived accuracy of (1) inaccurate and (2) accurate cancer news headlines, and (3) accuracy discernment (Table D.3), as well as intentions to share (4) inaccurate and (5) accurate cancer news and (6) sharing discernment (Table D.4). As Table D.3 (column 1) shows, self-reporting greater “low end” digital literacy associated with increased perceived accuracy of inaccurate headlines (*b* = 0.22, *SE* = 0.17, *p* = .006). This association is robust to the inclusion of a full set of predictors (column 2, *b* = 0.17, *SE* = 0.06, *p* = .007). “Low end” digital literacy is also associated with worse accuracy discernment when including the full set of predictors (*b* = *−*0.14, *SE* = 0.07, *p* = .037). As Table D.4 (column 1) shows, “low end” digital literacy is associated with greater intent to share inaccurate headlines (*b* = 0.28, *SE* = 0.11, *p* = .013), but this is not robust to the inclusion of additional predictors. We found no other associations of digital or health literacy in these sets of models.

**Fig. 3.**
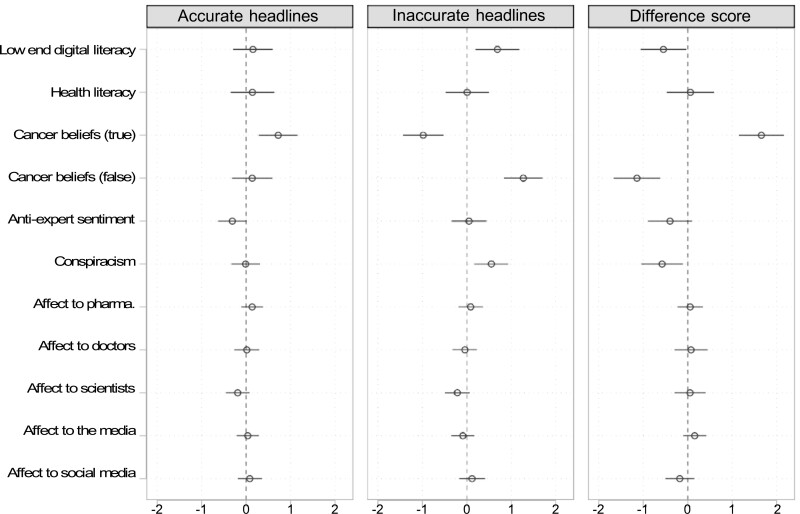
Correlates of perceived accuracy and discernment. Data come from the control group. All predictors are re-scaled to range from 0 to 1 for comparison. Perceived accuracy is measured on a scale that ranges from 1 to 4. All models include controls for age, female, college, nonwhite, and cancer history. Headline-level analyses include headline fixed effects.

Next, we modeled our outcome measures using a full set of predictors. For illustration, full models for perceived accuracy outcomes are shown in Fig. 3, with predictors re-scaled to range from 0 to 1 for direct comparability. This figure depicts point estimates from Table D.3 with 95% confidence intervals. In these models, conspiracism is associated with greater perceived accuracy of inaccurate headlines, worse accuracy discernment, and greater sharing intent for inaccurate headlines. Warmer affect toward pharmaceutical companies is associated with greater sharing intent for accurate headlines and better sharing discernment. Warmer affect toward the news media is associated with greater sharing intent for both accurate and inaccurate headlines. But the strongest, and most consistent predictors of these outcomes are pre-existing beliefs about cancer risk factors. False cancer beliefs are associated with greater perceived accuracy for inaccurate headlines, worse accuracy discernment, and greater sharing intent for inaccurate headlines. True cancer beliefs are associated with lower perceived accuracy for inaccurate headlines, greater perceived accuracy for accurate ones, better accuracy discernment, and greater sharing intent for accurate headlines. These associations are not negligible. For instance, the full model accounts for about 52% of the variance in accuracy discernment, an increase from 10% in the initial (literacy scales + controls) model. Given the importance of cancer risk factor beliefs in predicting news evaluations, we present exploratory models of these beliefs in Tables D.17 and D.18.

### Intervention Effects on Perceived Accuracy

We tested the main effects of the interventions across our outcome measures using a series of OLS regressions (Tables 3, 4 and Fig. D.2, which includes 95% confidence intervals). We found that the News Tips intervention had no effect on perceived accuracy outcomes. Meanwhile, the BOAST intervention decreased perceived accuracy of both inaccurate (*b* = *−*0.17, *SE* = 0.06, *p* = .007) and accurate cancer news headlines (*b* = −0.16, *SE* = 0.05, *p* = .001), but had no effect on accuracy discernment. We then calculated the difference in treatment effects between conditions to test whether BOAST’s effect was significantly larger than the News Tips’. Although the difference in effect on inaccurate headlines was not significant, BOAST’s effect on perception of accurate headlines was significantly more negative than the News Tips’ (*b* = −0.17, *SE* = 0.05, *p* = .001).

**Table 3:** Intervention Effects on Perceived Accuracy

	Inaccurate headlines	Accurate headlines	Difference score
News Tips	−0.1293 (0.0665)	0.0078 (0.0515)	0.1296 (0.0750)
BOAST	−0.1672** (0.0615)	−0.1645*** (0.0503)	−0.0043 (0.0695)
Constant	2.2042*** (0.0705)	2.6924*** (0.0522)	0.6536*** (0.0508)
Headline fixed effects	✓	✓	
BOAST—News Tips	−0.0379 (0.0639)	−0.1722*** (0.0519)	−0.1339 (0.0728)
N	3556	6180	593

^*^ *p* < .05, ** *p* < .01, *** *p* < .005 (two-sided). Cell entries are OLS coefficients with robust standard errors in parentheses.

**Table 4:** Intervention Effects on Sharing Intent

	Inaccurate headlines	Accurate headlines	Difference score
News Tips	−0.3099*** (0.0756)	−0.1665* (0.0806)	0.1457** (0.0546)
BOAST	−0.1784^*^ (0.0800)	−0.0990 (0.0819)	0.0912 (0.0522)
Constant	1.8593*** (0.0771)	2.3358*** (0.0882)	0.1417*** (0.0366)
Headline fixed effects	✓	✓	
BOAST—News Tips	0.1315 (0.0725)	0.0674 (.0781)	−0.0545 (0.0550)
*N*	3640	6185	T607

^*^ *p* < .05, ** *p* < .01, *** *p* < .005 (two-sided). Cell entries are OLS coefficients with robust standard errors in parentheses.

### Intervention Effects on Sharing Intent

Regarding sharing outcomes, conversely, we found that the News Tips intervention significantly reduced intent for inaccurate (*b* = *−*0.31, *SE* = 0.08, *p <* .001) and to a lesser extent accurate headlines (*b* = −0.17, *SE* = 0.08, *p* = .039), resulting in improved sharing discernment (*b* = 0.15, *SE* = 0.05, *p* = .008). BOAST on the other hand slightly decreased sharing intent for inaccurate headlines (*b* = 0.18, *SE* = 0.08, *p* = .026), but did not produce a significant improvement in sharing discernment.

### Magnitude of Intervention Effects on Perceived Accuracy and Sharing Intent

**Fig. 4.**
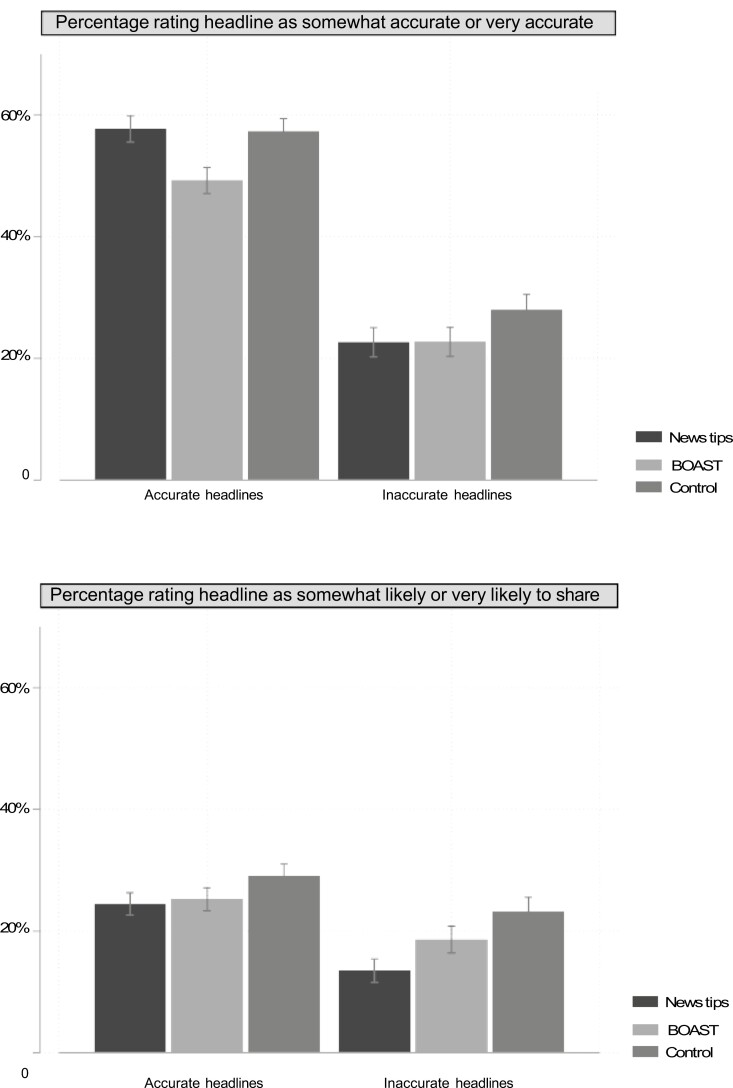
Intervention effects on accurate and inaccurate headlines (binary outcomes). Error bars are 95% confidence intervals of the mean.

We present standardized effect sizes (Cohen’s *d*) for simple treatment group differences versus the control in Table 5. Additionally, as seen in Tables D.5 and D.6 and depicted in Figure 4 with 95% confidence intervals, we illustrate the substantive magnitude of the intent to treat effects of the interventions using a binary indicator of perceived headline accuracy and a binary indicator of sharing intent. The proportion of respondents rating a false headline as “very accurate” or “somewhat accurate” decreased from 28% in the control condition to 23% among respondents who were assigned to the News Tips intervention, a difference of 5 percentage points. This effect represents a relative decrease of approximately one-fifth in the percentage of people wrongly endorsing inaccurate headlines. Meanwhile, the News Tips intervention did not decrease the proportion of those endorsing accurate headlines (57% in both treatment and control groups). Conversely, BOAST decreased both the proportion of those endorsing inaccurate headlines (to 23%, a 5% point difference to the control group) and the proportion of those endorsing accurate ones (to 49%, an 8 percentage point difference).

**Table 5:** Effect Sizes (Cohen’s *d*) for Treatment Group Differences Versus Control

	Inaccurate	Accurate	Difference score
		**Perceived Accuracy**	
BOAST versus control	−.18 [−.10, −.26]	−.19 [−.13, −.26]	−.01 [−.20, .19]
News Tips versus control	−.13 [−.05, −.21]	.00 [−.06, .06]	.17 [.02, .37]
		**Sharing intent**	
BOAST versus control	−.18 [−.10, −.26]	−.09 [−.03, −.15]	.17 [−.02, .37]
News Tips versus control	−.33 [−.25, −.41]	−.16 [−.10, −.23]	.26 [.07, .46]

Note: 95% CIs in brackets.

Turning to the binary indicators for sharing intent, the News Tips intervention decreased the proportion “somewhat” or “very likely” to share for both inaccurate (from 23% to 14%, a 9% point difference) and to a lesser extent accurate headlines (from 28% to 24%). The BOAST intervention’s effects were smaller in magnitude (a 1% point difference for inaccurate headlines and a 3% point difference for accurate ones).

### Heterogeneous Treatment Effects

Finally, we test for differential intervention effects across predictors tested above (cancer beliefs [true], cancer beliefs [false], antiexpert sentiment, conspiracism, and affect toward pharmaceuticals, doctors, scientists, the media, and social media) as well as personal cancer history. Full model results are reported in the Appendix by outcome (Tables D.9 through D.14). We found limited evidence of differential effects across these predictors with the exception of pre-existing cancer beliefs.

**Fig. 5.**
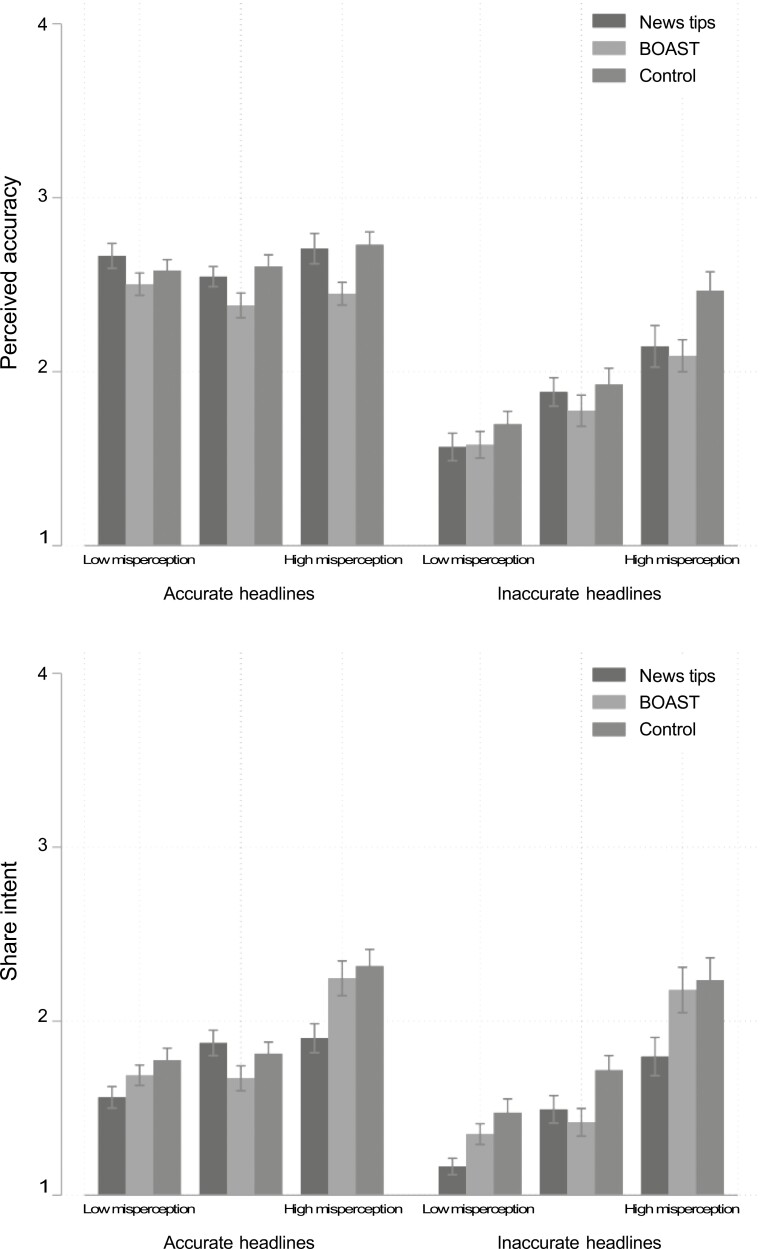
Intervention effects on accurate and inaccurate headlines across pre-treatment mis-perceptions about cancer (terciles). Error bars are 95% confidence intervals of the mean.

Focusing on these models (Tables D.15, D.16, and Fig. 5, featuring 95% confidence intervals), we find that false cancer beliefs in particular moderate the effects of both interventions on perceived accuracy of inaccurate headlines as well as accuracy discernment, such that those holding greater belief in cancer misperceptions experienced greater decreases in perceived accuracy of inaccurate headlines and greater increases in discernment. A similar pattern holds for sharing outcomes. An inspection of Fig. 5 suggests that these relatively larger gains may be due in part to the fact that high-misperception respondents in the control condition tended to give the highest accuracy ratings and express the greatest sharing intentions for inaccurate headlines, meaning they had more misclassifications to correct via treatment.

## Discussion

Prior research found media literacy tips interventions decrease belief in dubious political news, but these had not been tested with health news specifically. We tested the viability of a novel, health-focused set of media literacy tips (BOAST) against an established generic media literacy (News Tips) intervention as a baseline. Ultimately, our findings suggest the BOAST intervention resulted in complicated effects: The intervention led to no improvement in the discernment of accurate/inaccurate cancer news, as it drove down perceived accuracy ratings of legitimate news stories *in addition to* driving down accuracy ratings of inaccurate news stories.

Although we cannot state definitively which components of the BOAST intervention precipitated these less-than-ideal outcomes, we offer some speculation. It is possible that because respondents in the BOAST condition were told to be wary of research too early to be useful, they somewhat discounted many accurate cancer news headlines covering promising early-stage studies with prospective language (e.g., “Research shows promising results for future of cancer treatments through vaccine,” “Breast density changes over time could be linked to breast cancer risk, study finds,” and “Blood-test biopsy could speed up cancer treatment”), especially as they could not access the full article for context. Other accurate headlines featuring pharmaceutical companies may have been discounted due to the warnings regarding sales-focused content or bias more generally (e.g., “AI vs. cancer: AstraZeneca exec reveals how COVID pandemic helped develop early cancer diagnosis tech”).

In addition to the unintended effect of increasing skepticism of accurate information, several limitations should be offered. First, the effect sizes were modest; simple interventions did not fully eliminate belief in inaccurate headlines, for instance. Second, we did not examine the degree to which effects decayed over time. This, along with other design specifications, would allow us to tease apart effect mechanisms (be they the result of increased accuracy concerns or the result of learned [and applied] heuristics). Third, we did not observe potential changes in real-world behavior downstream from exposure. Finally, we did not test the entire BOAST intervention—which includes providing participants feedback and examples. Conclusions about that tool are limited here to the high-line information from BOAST rather than the full intervention as offered currently online.

That said, our results underscore the importance of testing—for either novel interventions, or for established ones being extended to new contexts—as the pattern of results we observed would not be directly predictable by relying on prior studies. Based on the spillover effects detected for the novel BOAST intervention, future work can help improve this tip set in a few ways. Future iterations should consider and build on recent calls for interventions that mix both typical skepticism-inducing tips with those that induce *trust* for legitimate sources of information [13]. Trust-inducing tips have shown promise in initial work but should also be tested in the health news domain specifically. In addition, future iterations can make use of “unbundling” to better understand the discrete effects of each individual tip in the bundle tested here [39]. This could allow for an optimized bundle of tips by removing those most detrimental to legitimate news.

Finally, our findings regarding pre-existing cancer beliefs can be informative for intervention design. False cancer beliefs are the strongest predictor of poor accuracy discernment, and individuals with greater levels of cancer misperceptions benefit most from the interventions. These findings can be useful in a few ways, then. First, future interventions can target these common misperceptions directly—i.e., through simple informational or corrective interventions concerning cancer risks—alone or in combination with providing more general health news evaluation advice (though, of course, some of these misperceptions might require more than a simple correction). Further, because common markers of cancer disparities like race, education, and income [40] are associated with these misperceptions (as shown in Table D.18), such interventions may help to address these disparities, if indirectly. Second, it may be most resource-efficient to target those higher in cancer misperceptions as they are both most at risk to believe and share inaccurate cancer news and most improved by exposure to interventions [41].

## Conclusion

In sum, we contribute to the literature on applied media literacy by comparing the effects of generic and domain-specific interventions for the first time, with a novel focus on cancer news content evaluations. We show that the generic media literacy intervention’s efficacy can indeed generalize across news domains. However, we show that the health-focused media literacy intervention we tested raised suspicion of accurate content, in line with past work showing that misinformation- focused interventions can often backfire, necessitating the inclusion trust-inducing tips as well [13]. Lastly, we contribute a better understanding of the prominent role played by pre-existing domain-specific misperceptions in (a) predicting perceived accuracy of and engagement with inaccurate news content and (b) conditioning efficacy of treatment. Those most misinformed about cancer benefited the most from these brief interventions. As such, we build on prior work [26] to suggest a more fruitful approach to interventions going forward.

## Supplementary Material

Supplementary material is available at *Annals of Behavioral Medicine* online.

kaae054_suppl_Supplementary_Material

## OSF Registration

https://osf.io/984t5/?view_only=b4453e6cc1d643cfa3562a20fd9e648c

## Transparency Statements

(1) The study was pre-registered at https://osf.io/984t5/?view_only=b4453e6cc1d643cfa3562a20fd9e648c
(2) The analysis plan was formally registered at https://osf.io/984t5/?view_only=b4453e6cc1d643cfa3562a20fd9e648c
(3) De-identified data from this study are available at https://osf.io/nsy87/?view_only=f37575219b56483bba395f45771c94ca
(4) Analytic code used to conduct the analyses presented in this study are available in the public archive https://osf.io/nsy87/?view_only=f37575219b56483bba395f45771c94ca
(5) Materials used to conduct the study are publicly available at https://osf.io/nsy87/?view_only=f37575219b56483bba395f45771c94ca
