# Supplementary material for: A Health Media Literacy Intervention Increases Skepticism of Both Inaccurate and Accurate Cancer News Among U.S. Adults
Source: Ann Behav Med. 2024 Sep 26;58(12):820–31. doi: 10.1093/abm/kaae054 (PMC11568353; doi:10.1093/abm/kaae054)
Supplement: kaae054_suppl_Supplementary_Material [file kaae054_suppl_supplementary_material.pdf]

A Methods detail

We draw on data from a survey conducted among a representative sample of the U.S. population by the survey company YouGov, which recruits a large panel of opt-in respondents and then uses a weighting and matching algorithm to construct a final sample that mirrors the demographic composition of the U.S. population. Data come from a study fielded October 25 - November 21, 2023.

Specifically, YouGov interviewed 1325 respondents who were then matched down to a sample of 1200 to produce the final dataset. The respondents were matched to a sampling frame on gender, age, race, education, and treatment received. The sampling frame is a politically representative "modeled frame" of US adults, based upon the American Community Survey (ACS) public use microdata file, public voter file records, the 2020 Current Population Survey (CPS) Voting and Registration supplements, the 2020 National Election Pool (NEP) exit poll, and the 2020 CES surveys, including demographics and 2020 presidential vote.

The matched cases were weighted to the sampling frame using propensity scores. The matched cases and the frame were combined and a logistic regression was estimated for inclusion in the frame. The propensity score function included age, gender, race/ethnicity, years of education, home ownership status (own, rent, other), 2020 presidential vote, and region. The propensity scores were grouped into deciles of the estimated propensity score in the frame and post-stratified according to these deciles. The weights were then post-stratified on 2020 presidential vote choice, home ownership status, region (4-categories), as well as a four-way stratification of gender, age (4-categories), race (4-categories), and education (4-categories), to produce the final weight.

B News headline stimuli

Note: The full set of headline images used the study can be accessed here: https://osf.io/nsy87/?view_only=f37575219b56483bba395f45771c94ca

Inaccurate headlines and urls

Top Scientists Find 'Substantial Scientific Evidence' RF Radiation Causes Cancer
https://childrenshealthdefense.org/defender/radiofrequency-rf-radiation-cancer/

Top Pathologist confirms Cancer, Infertility & Strange Blood Clots are common side effects of Covid-19 Vaccination
https://expose-news.com/2023/04/22/cancer-infertility-blood-clots-due-to-covid-vaccination/

The COVID-19 mRNA "Vaccines" cause Cancer; here's the evidence…
https://expose-news.com/2022/08/02/the-covid-19-mrna-vaccines-cause-cancer/

Are COVID Boosters Behind Increase in Cancer Among Younger Adults?
https://childrenshealthdefense.org/defender/covid-boosters-increase-cancer-young-adults-cola/

THIS SMALL FRUIT IS A REAL TREASURE: CAN CURE PROSTATE CANCER AND PREVENT STOMACH AND COLON CANCER!
https://instiks.com/this-small-fruit-is-a-real-treasure-can-cure-prostate-cancer-and-prevent-stomach-and-colon-cancer/

50-Year-Old Man Cures Lung Cancer With Cannabis Oil, Stuns CBS News
http://4healthmm.com/50-year-old-man-cures-lung-cancer-cannabis-oil-stuns-cbs-news/#:~:text=4%20Health%20MM-,50%2DYear%2DOld%20Man%20Cures%20Lung%20Cancer%20With,Cannabis%20Oil%2C%20Stuns%20CBS%20News&text=A%20combination%20of%20cannabis%20oil,others%20heal%20their%20lives%2C%20too.

Kashmiri scientist discovers prostate cancer treatment
https://www.greaterkashmir.com/todays-paper/kashmiri-scientist-discovers-prostate-cancer-treatment

Cow urine cured my breast cancer: Sadhvi Pragya
https://www.indiatoday.in/elections/lok-sabha-2019/story/sadhvi-pragya-cow-urine-cancer-1507816-2019-04-22

Women recently injected with experimental covid vaccines are showing symptoms of BREAST CANCER
https://dcdirtylaundry.com/women-recently-injected-with-experimental-covid-vaccines-are-showing-symptoms-of-breast-cancer/

Cancer Is a Fungus, Cancer Is a Frequency: Dr. Darrell Wolfe
https://thesternmethod.com/darrell-wolfe/

The Cancer Industry is too prosperous to allow a cure
https://www.naturalnews.com/055697_cancer_industry_big_pharma_natural_cures.html

Cover-Up Of Promising Cancer Treatment | Cancer Research | Documentary
https://www.youtube.com/watch?v=ee9KCGZvVfA

Top 7 reasons chemotherapy fails more than 97% of the time, creating new cancers in the body and crippling the chances of true recovery
https://www.naturalnews.com/054324_cancer_chemotherapy_natural_cures.html

The Sugar And Cancer Connection
https://natureworksbest.com/sugar-feeds-cancer-growth/

Can cancer really be cured with ivermectin and other safe, old treatments?
https://drtesslawrie.substack.com/p/can-cancer-really-be-cured-with-ivermectin/comments

Man With "Terminal" Lung Cancer Cures Himself With Cannabis Oil
https://www.thehealthcure.org/blog/man-with-terminal-lung-cancer-cures-himself-with-cannabis-oil/

Accurate headlines and urls

Video distraction helps kids undergo cancer radiotherapy, Stanford Medicine-led study finds
https://med.stanford.edu/news/all-news/2023/05/kids-cancer-video-distraction.html

Gene-editing technique could speed up study of cancer mutations
https://news.mit.edu/2023/gene-editing-technique-cancer-mutations-0511

Antibiotics after breast cancer linked to poorer survival, Stanford Medicine-led study finds
https://med.stanford.edu/news/all-news/2023/04/antibiotics-cancer.html

Breast density changes over time could be linked to breast cancer risk, study finds
https://www.cnn.com/2023/05/02/health/breast-density-cancer-study/index.html

Vaping may 'wake up' cancer cells and trigger wave of disease in a decade
https://www.independent.co.uk/life-style/health-and-families/health-news/vaping-ecigarettes-cancer-research-b2165228.html

Very few Americans know drinking alcohol increases cancer risk, study finds
https://www.today.com/health/doctors-sound-alarm-alcohol-cancer-link-study-finds-americans-aware-rcna59696

Research shows promising results for future of cancer treatments through vaccines
https://abcnews.go.com/Health/video/research-shows-promising-results-future-cancer-treatments-vaccines-99254988

You don't need to walk 10,000 steps a day — walking faster is what counts to protect you from heart disease and cancer
https://news.yahoo.com/dont-walk-10-000-steps-170514582.html

Health panel recommends women get screening mammograms at age 40
https://www.washingtonpost.com/wellness/2023/05/09/mammogram-age-40-breast-cancer-screening/

A Scary New Link Between Air Pollution and Lung Cancer
https://www.washingtonpost.com/business/a-scary-new-link-between-air-pollution-and-lung-cancer/2022/09/15/91654f22-350f-11ed-a0d6-415299bfebd5_story.html

Link Between Long Telomeres and Long Life Is a Tall Tale, Study Finds
https://www.nytimes.com/2023/05/04/health/long-telomeres-age-longevity.html

Sugary drinks associated with increased risk of death from cancer
https://www.msn.com/en-us/health/medical/sugary-drinks-associated-with-increased-risk-of-death-from-cancer/ar-AA11RXmm

Genetic test for cancer is less accurate for Black and Asian people
https://www.msn.com/en-us/health/medical/genetic-test-for-cancer-is-less-accurate-for-black-and-asian-people/ar-AA12peqj

New mRNA Pancreatic Cancer Vaccine Trial Starts Next Phase
https://www.mskcc.org/news/can-mrna-vaccines-fight-pancreatic-cancer-msk-clinical-researchers-are-trying-find-out

For Advanced Endometrial Cancer, Chemotherapy Plus Immunotherapy Improves Outcomes
https://www.mskcc.org/news/advanced-endometrial-cancer-chemotherapy-plus-immunotherapy-improves-outcomes

Screen all women for breast cancer at 40, instead of 50, new guidelines say
https://www.nbcnews.com/health/womens-health/breast-cancer-guidelines-start-screenings-age-40-rcna83355

Pancreatic Cancer Vaccine Shows Promise in Small Trial
https://www.nytimes.com/2023/05/10/health/pancreatic-cancer-vaccine-mrna.html

An mRNA vaccine that programs the body to fight pancreatic cancer shows early promise
https://www.cnn.com/2023/05/10/health/pancreatic-cancer-vaccine/index.html

Rare Melanoma Very Likely to Respond to Treatment with Pembrolizumab
https://www.cancer.gov/news-events/cancer-currents-blog/2023/pembrolizumab-alone-desmoplastic-melanoma

Too Many Older Men Are Still Screened for Prostate Cancer
https://www.nytimes.com/2023/05/08/health/prostate-cancer-screening.html?auth=login-email&login=email&searchResultPosition=1

AI vs. cancer: AstraZeneca exec reveals how COVID pandemic helped develop early cancer diagnosis tech
https://www.foxnews.com/media/ai-cancer-astrazeneca-exec-covid-pandemic-helped-tech-early-cancer-diagnosis

Cancer Research Points to Key Unknowns about Popular "Antiaging" Supplements
https://www.scientificamerican.com/article/cancer-research-points-to-key-unknowns-about-popular-antiaging-supplements/

Transgender women on hormones face higher risk of prostate cancer going undiagnosed: Study
https://www.foxnews.com/media/transgender-women-hormones-higher-risk-prostate-cancer-undiagnosed-study

Dermatologists found the ’world’s smallest skin cancer’ under a woman’s eye — it was smaller than the tip of a pen
https://www.msn.com/en-us/health/medical/dermatologists-found-the-world-s-smallest-skin-cancer-under-a-woman-s-eye-it-was-smaller-than-the-tip-of-a-pen/ar-AA1aHaVm

Artificial intelligence helping detect early signs of breast cancer in some US hospitals
https://www.msn.com/en-us/health/medical/artificial-intelligence-helping-detect-early-signs-of-breast-cancer-in-some-us-hospitals/ar-AA1aFDMU

Inhaled steroids for asthma may offer some cancer protection, study says
https://www.washingtonpost.com/wellness/2023/05/01/asthma-cancer-steroids/

Change in Oral Sex Attitudes Causing Rise in HPV-Related Throat Cancer
https://www.newsweek.com/oral-sex-hpv-infections-increasing-throat-cancer-1797374

Love that ‘new car smell’? Study says there are cancer-causing chemicals to consider
https://www.foxnews.com/health/love-new-car-smell-study-says-there-are-cancer-causing-chemicals-consider

Blood-test biopsy could speed up cancer treatment
https://www.msn.com/en-xl/news/other/blood-test-biopsy-could-speed-up-cancer-treatment/ar-AA1aiETu

Cancer survivors may be at heightened risk of cardiovascular disease
https://www.theguardian.com/society/2023/apr/18/cancer-survivors-heightened-risk-heart-cardiovascular-disease

Liquid Biopsies on the Horizon for Children with Solid Cancer
https://www.cancer.gov/news-events/cancer-currents-blog/2023/liquid-biopsy-children-solid-cancers

EPA proposes first-ever standard for cancer causing chemicals in drinking water
https://www.foxnews.com/politics/epa-proposes-first-ever-limit-cancer-causing-chemicals-drinking-water

Artificial turf potentially linked to cancer deaths of six Phillies ball players – report
https://www.theguardian.com/society/2023/mar/10/phillies-ball-players-cancer-artifical-turf

Cancer will cost the world $25 trillion over next 30 years
https://www.nature.com/articles/d41586-023-00634-9

After 30 years of research, pill developed for breast cancer approved for use
https://www.seattletimes.com/seattle-news/health/after-30-years-of-research-pill-developed-for-breast-cancer-approved-for-use/

An ant’s sense of smell is so strong, it can sniff out cancer
https://www.washingtonpost.com/climate-environment/2023/01/24/ants-smell-cancer/

Child cancer rates higher than normal in New Jersey town with contaminated water
https://www.foxnews.com/us/child-cancer-rates-higher-normal-new-jersey-town-c

ontaminated-water

Cancer Vaccine Created via CRISPR Prevents and Stops Brain Tumors
https://www.psychologytoday.com/us/blog/the-future-brain/202301/cancer-vaccine-created-via-crispr-prevents-and-stops-brain-tumors

'Overwhelming' response for radon testing after Lehi woman shares cancer diagnosis story
https://www.ksl.com/article/50561517/overwhelming-response-for-radon-testing-after-lehi-woman-shares-cancer-diagnosis-story

Patients with cancer at greater risk of suicide
https://www.upi.com/Health_News/2023/01/23/cancer-diagnosis-suicide-risk/2281674231882/

Pancreatic Cancer Vaccine Shows Promise in Small Initial Trial
https://www.scientificamerican.com/article/pancreatic-cancer-vaccine-shows-promise-in-small-initial-trial/

Safest sunscreens to use this summer, according to experts
https://www.cnn.com/2023/05/23/health/sunscreen-guide-2023-wellness/index.html

"Radioactive" roads made of potentially cancer-causing mining waste could be headed to Florida under new bill
https://www.cbsnews.com/news/radioactive-roads-phosphogypsum-mining-could-be-headed-to-florida/

Cancer Vaccines Poised to Unlock 'New Treatment Paradigm' With Merck/Moderna Data
https://www.usnews.com/news/top-news/articles/2023-06-05/cancer-vaccines-poised-to-unlock-new-treatment-paradigm-with-merck-moderna-data

C Survey question wording

Pre-treatment measures

Note: We use demographic measures as provided by YouGov.

Health insurance
Do you currently have health insurance?
• Yes, I have private insurance (for example, through my job, HMO)
• Yes, I have public insurance (for example, Medicaid, Medicare)
• No

Binary cancer history indicator
Have you ever been diagnosed with cancer?
• Yes
• No
• [If yes] What type? [open]

Frequency of social media usage
How frequently do you use social media?
• Almost constantly
• Several times a day
• About once a day
• About once a week
• A few times a month
• Once a month
• Less often than once a month
• Never

Anti-expert views
Average of 3 items, 5 pt Likert (agree/disagree):
• I am more confident in my opinion than other people's facts
• Most of the time I know just as much as experts
• Experts really don't know that much

Conspiracism
Average of 4 items, 5 pt Likert (agree/disagree):
• Much of our lives are being controlled by plots hatched in secret places.
• Even though we live in a democracy, a few people will always run things anyway.
• The people who really 'run' the country are not known to the voter.
• Big events like wars, recessions, and the outcomes of elections are controlled by small groups of people who are working in secret against the rest of us.

"Low-end" digital literacy scale
Average of 5 items, (Never/Almost never/Occasionally/Somewhat often/Very often):
• I rely on family members to introduce me to new technology.
• I have professionals (such as the Geek Squad) or family members take a look at my computer when something isn't working.
• A lot of the things I see online confuse me.
• I have problems with viruses and malware on my computer.
• I have trouble finding things that I've saved on my computer.

Brief Health Literacy Screener

Average of 4 items
(Always, Often, Sometimes, Occasionally, Never):
• How often do you have someone help you read hospital materials?
• How often do you have problems learning about your medical condition because of difficulty understanding written information?
• How often do you have a problem understanding what is told to you about your medical condition?
(Not at all, A little bit, Somewhat, Quite a bit, Extremely):
• How confident are you filling out medical forms by yourself?

Feeling Thermometers
(1-100 sliders)
Next, we would like to know your feelings toward some groups of people using something we call the feeling thermometer. Ratings between 50° and 100° mean that you feel favorable and warm toward the person or the group of people. Ratings between 1° and 50° mean that you don't feel favorable toward the person or group of people and that you don't care too much for them. You would rate the person or group of people at the 50° mark if you don't feel particularly warm or cold toward them.
• Pharmaceutical companies
• Doctors
• News media
• Scientists in general
• Social media

Cancer Perceptions Battery
5 pt Likert (agree/disagree, "not sure" as the midpoint).
How much do you agree that each of these can increase a person's chance of developing cancer?
• Exposure to non-ionizing electromagnetic frequencies (WiFi, 5G, radio)*
• Feeling stressed*
• Using cleaning products*
• Eating genetically modified food*
• Getting a COVID-19 vaccine*
• Using microwave ovens*
• Drinking from plastic bottles*
• Drinking more than 1 unit of alcohol a day
• Being overweight or obese (BMI over 25)
• Getting sunburnt more than once as a child
• Being over 70 years old
• Having a close relative with cancer
• Infection with HPV (human papillomavirus)
• Doing less than 30 minutes of physical activity 5 times a week

Outcome measures

Perceived accuracy
4 pt. ("Not at all accurate" (1) to "Very accurate" (4))
To the best of your knowledge, how accurate is the claim in the above headline?

Sharing intent
4 pt. ("Not at all likely" (1) "Very likely" (4))
How likely would you be to share this story online (for example, through Facebook or Twitter/X?)

D Additional results

Table D.1: Balance tests (pt. 1)

	Age	Female	Nonwhite	College	Cancer	Dig. lit	Health lit	Anti-expert	Conspiracy
BOAST	0.5100	0.0175	0.0350	0.0575	-0.0175	-0.0044	0.0394	-0.0633	-0.0400
	(1.1066)	(0.0353)	(0.0313)	(0.0347)	(0.0229)	(0.0489)	(0.0490)	(0.0623)	(0.0717)
News tips	-0.2300	0.0100	0.0825*	0.0100	-0.0125	0.0206	0.0037	0.0075	-0.0175
	(1.1191)	(0.0353)	(0.0320)	(0.0344)	(0.0231)	(0.0493)	(0.0500)	(0.0627)	(0.0724)
Constant	51.5175***	0.5225***	0.2500***	0.3775***	0.1275***	1.9794***	4.4031***	2.6417***	3.0606***
	(0.7579)	(0.0250)	(0.0217)	(0.0243)	(0.0167)	(0.0328)	(0.0346)	(0.0451)	(0.0509)
R^2	0.00	0.00	0.01	0.00	0.00	0.00	0.00	0.00	0.00
N	1200	1200	1200	1200	1200	1200	1200	1200	1200

* $p < .05$, ** $p < .01$, *** $p < .005$ (two-sided). Cell entries are OLS coefficients.

Table D.2: Balance tests (pt. 2)

	Pharma FT	Media FT	Scientist FT	SM FT	Dr. FT	SM use	Cancer (true)	Cancer (false)
BOAST	0.3529	-0.4425	1.8375	1.7850	2.0575	0.0200	0.0989*	0.0357
	(1.8987)	(1.9692)	(1.9386)	(1.8451)	(1.8133)	(0.1445)	(0.0493)	(0.0513)
News tips	-1.2350	-0.1775	-0.0525	0.6750	-0.5300	0.1075	0.0200	0.0804
	(1.9377)	(2.0586)	(2.0198)	(1.8623)	(1.8741)	(0.1517)	(0.0483)	(0.0507)
Constant	41.2141***	42.4450***	59.2100***	38.0850***	60.5950***	3.0600***	3.2786***	2.6321***
	(1.3677)	(1.4188)	(1.4071)	(1.2923)	(1.3121)	(0.1014)	(0.0354)	(0.0352)
R^2	0.00	0.00	0.00	0.00	0.00	0.00	0.00	0.00
N	1154	1200	1200	1200	1200	1200	1200	1200

* $p < .05$, ** $p < .01$, *** $p < .005$ (two-sided). Cell entries are OLS coefficients.

Table D.3: Correlates of perceived accuracy and discernment

	Inaccurate headlines		Accurate headlines		Difference score	
"Low end" digital literacy	0.2218**	0.1718**	0.0639	0.0386	-0.1511	-0.1364*
	(0.0803)	(0.0633)	(0.0573)	(0.0569)	(0.0798)	(0.0651)
Health literacy	-0.0741	0.0019	0.0524	0.0356	0.1204	0.0156
	(0.0714)	(0.0626)	(0.0567)	(0.0627)	(0.0669)	(0.0680)
Cancer beliefs (true)		-0.2453***		0.1806***		0.4143***
		(0.0583)		(0.0558)		(0.0649)
Cancer beliefs (false)		0.3173***		0.0344		-0.2853***
		(0.0551)		(0.0583)		(0.0671)
Anti-expert sentiment		0.0117		-0.0772		-0.0993
		(0.0502)		(0.0413)		(0.0636)
Conspiracism		0.1376**		-0.0027		-0.1439*
		(0.0490)		(0.0413)		(0.0602)
Affect toward pharmaceuticals		0.0009		0.0014		0.0006
		(0.0014)		(0.0013)		(0.0015)
Affect toward doctors		-0.0005		0.0002		0.0008
		(0.0014)		(0.0015)		(0.0020)
Affect toward scientists		-0.0022		-0.0019		0.0005
		(0.0015)		(0.0014)		(0.0018)
Affect toward the media		-0.0009		0.0004		0.0016
		(0.0014)		(0.0013)		(0.0014)
Affect toward social media		0.0012		0.0008		-0.0018
		(0.0015)		(0.0014)		(0.0017)
Age 30-44	0.6149***	0.2629*	0.3755***	0.3721**	-0.2578	0.1118
	(0.1466)	(0.1226)	(0.1272)	(0.1351)	(0.2009)	(0.1795)
Age 45-59	0.5876***	0.2385	0.3053*	0.3912***	-0.3035	0.1577
	(0.1407)	(0.1240)	(0.1215)	(0.1350)	(0.1956)	(0.1716)
Age 60+	0.3675**	-0.0396	-0.0268	0.0621	-0.4170*	0.1031
	(0.1401)	(0.1261)	(0.1281)	(0.1344)	(0.1979)	(0.1676)
Female	-0.1156	-0.1224	-0.1656*	-0.1750**	-0.0689	-0.0708
	(0.0836)	(0.0710)	(0.0660)	(0.0658)	(0.0991)	(0.0789)
College	-0.1881*	-0.0934	0.0383	-0.0365	0.2000	0.0421
	(0.0847)	(0.0668)	(0.0651)	(0.0639)	(0.1033)	(0.0849)
Nonwhite	0.1222	-0.0249	-0.0544	-0.0037	-0.1537	0.0546
	(0.0941)	(0.0793)	(0.0703)	(0.0736)	(0.1086)	(0.0887)
Cancer history	-0.0868	0.1192	0.0486	0.0184	0.1720	-0.0796
	(0.1280)	(0.1121)	(0.0970)	(0.0903)	(0.1258)	(0.0939)
Constant	1.4754***	1.2190**	2.1067***	1.7234***	0.7072	0.7351
	(0.3989)	(0.4470)	(0.2958)	(0.4098)	(0.3721)	(0.4577)
Headline fixed effects	✓	✓	✓	✓		
R^2	0.18	0.32	0.19	0.22	0.10	0.52
N	1188	1140	2069	1985	198	190

* $p < .05$, ** $p < .01$, *** $p < .005$ (two-sided). Cell entries are OLS coefficients. Data come from the control group.

Table D.4: Correlates of sharing intent and discernment

	**Inaccurate headlines**		**Accurate headlines**		**Difference score**	
"Low end" digital literacy	0.2801*	0.1445	0.1744	0.1080	-0.1043	-0.0270
	(0.1114)	(0.1008)	(0.1090)	(0.0945)	(0.0601)	(0.0608)
Health literacy	-0.0712	-0.0345	-0.0809	-0.0295	-0.0042	0.0121
	(0.1002)	(0.0939)	(0.1010)	(0.0986)	(0.0580)	(0.0567)
Cancer beliefs (true)		0.1157		0.2381*		0.1337
		(0.0948)		(0.0998)		(0.0690)
Cancer beliefs (false)		0.3000***		0.1748		-0.1279
		(0.0873)		(0.0955)		(0.0699)
Anti-expert sentiment		0.1681*		0.0969		-0.0684
		(0.0670)		(0.0769)		(0.0540)
Conspiracism		0.1624***		0.0947		-0.0716
		(0.0562)		(0.0593)		(0.0388)
Affect toward pharmaceuticals		0.0040		0.0067***		0.0034*
		(0.0021)		(0.0023)		(0.0017)
Affect toward doctors		-0.0007		-0.0009		-0.0008
		(0.0022)		(0.0025)		(0.0018)
Affect toward scientists		-0.0026		-0.0023		0.0007
		(0.0019)		(0.0022)		(0.0017)
Affect toward the media		0.0055**		0.0068***		0.0010
		(0.0021)		(0.0022)		(0.0016)
Affect toward social media		0.0007		-0.0003		-0.0013
		(0.0022)		(0.0023)		(0.0015)
Age 30-44	0.0779	0.0550	-0.1957	-0.1511	-0.2725	-0.2110
	(0.2344)	(0.2083)	(0.2549)	(0.2440)	(0.1676)	(0.1613)
Age 45-59	0.1564	0.1292	-0.2303	-0.1929	-0.4141*	-0.3618*
	(0.2438)	(0.2145)	(0.2621)	(0.2554)	(0.1770)	(0.1761)
Age 60+	-0.0289	0.0424	-0.2386	-0.1555	-0.2246	-0.2249
	(0.2417)	(0.2087)	(0.2663)	(0.2517)	(0.1851)	(0.1759)
Female	0.0593	0.1047	0.1916	0.1915	0.1292	0.0849
	(0.1155)	(0.1091)	(0.1220)	(0.1178)	(0.0735)	(0.0705)
College	-0.3468***	-0.2497*	-0.0991	-0.1119	0.2448***	0.1253
	(0.1101)	(0.1047)	(0.1183)	(0.1176)	(0.0832)	(0.0805)
Nonwhite	0.4005**	0.1952	0.3218*	0.1232	-0.1183	-0.1176
	(0.1530)	(0.1496)	(0.1468)	(0.1511)	(0.0830)	(0.0878)
Cancer history	0.0284	0.0384	0.2088	0.1792	0.1698	0.1299
	(0.1864)	(0.1630)	(0.1921)	(0.1782)	(0.0960)	(0.0984)
Constant	1.4752*	-0.8376	2.3228***	0.0053	0.4875	0.4854
Headline fixed effects	✓	✓	✓	✓		
	(0.5702)	(0.6759)	(0.5756)	(0.7287)	(0.3175)	(0.4622)
R^2	0.13	0.25	0.09	0.19	0.13	0.26
N	1211	1157	2069	1976	202	193

* $p < .05$, ** $p < .01$, *** $p < .005$ (two-sided). Cell entries are OLS coefficients. Data come from the control group.

Figure D.1: Histograms for average perceived accuracy and sharing intent outcomes in the control condition

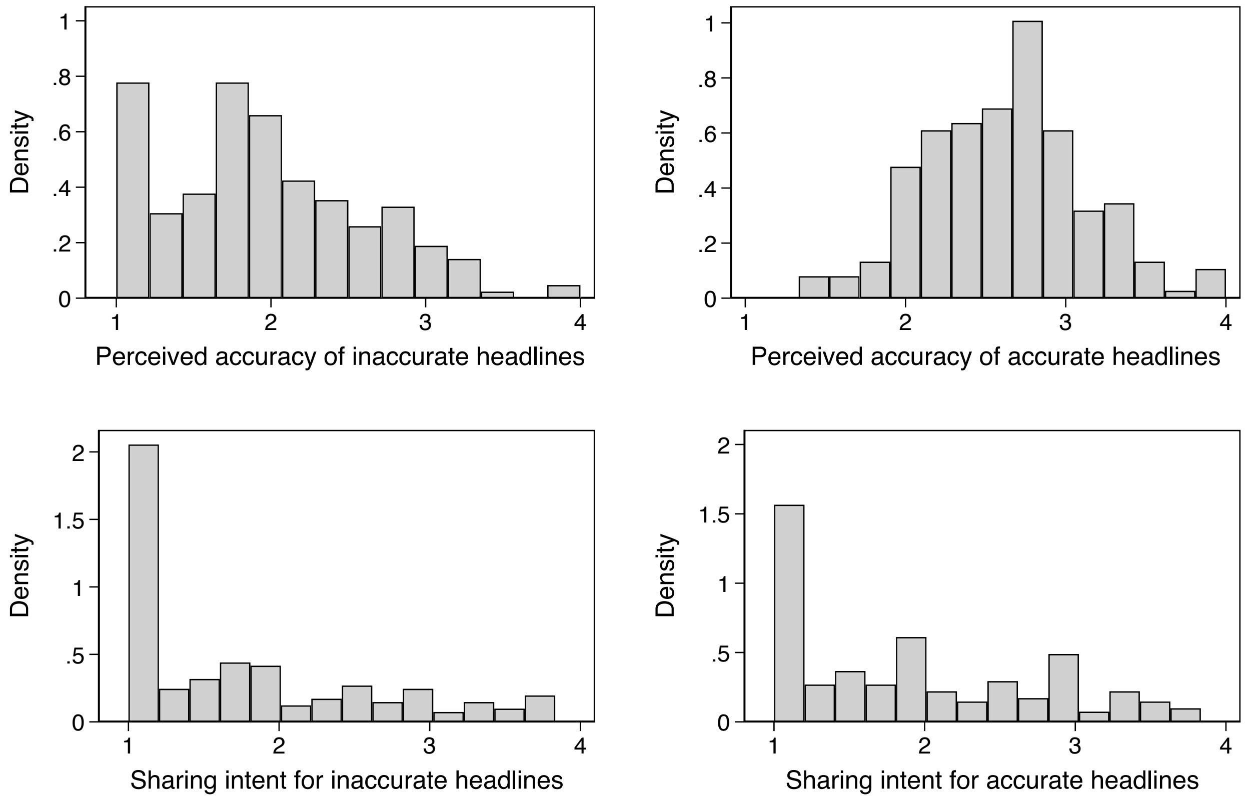

Table D.5: Intervention effects on perceived accuracy (binary)

	Inaccurate headlines	Accurate headlines	Difference score
News tips	-0.0560*	0.0065	0.0596
	(0.0282)	(0.0260)	(0.0345)
BOAST	-0.0542*	-0.0761***	-0.0264
	(0.0269)	(0.0264)	(0.0321)
Constant	0.3649***	0.6060***	0.2871***
	(0.0357)	(0.0287)	(0.0237)
Headline fixed effects	✓	✓	
BOAST - news tips	0.0018	-0.0826***	-0.0860**
	(0.0268)	(0.0266)	(0.0332)
N	3556	6180	593

* $p < .05$, ** $p < .01$, *** $p < .005$ (two-sided). Cell entries are OLS coefficients.

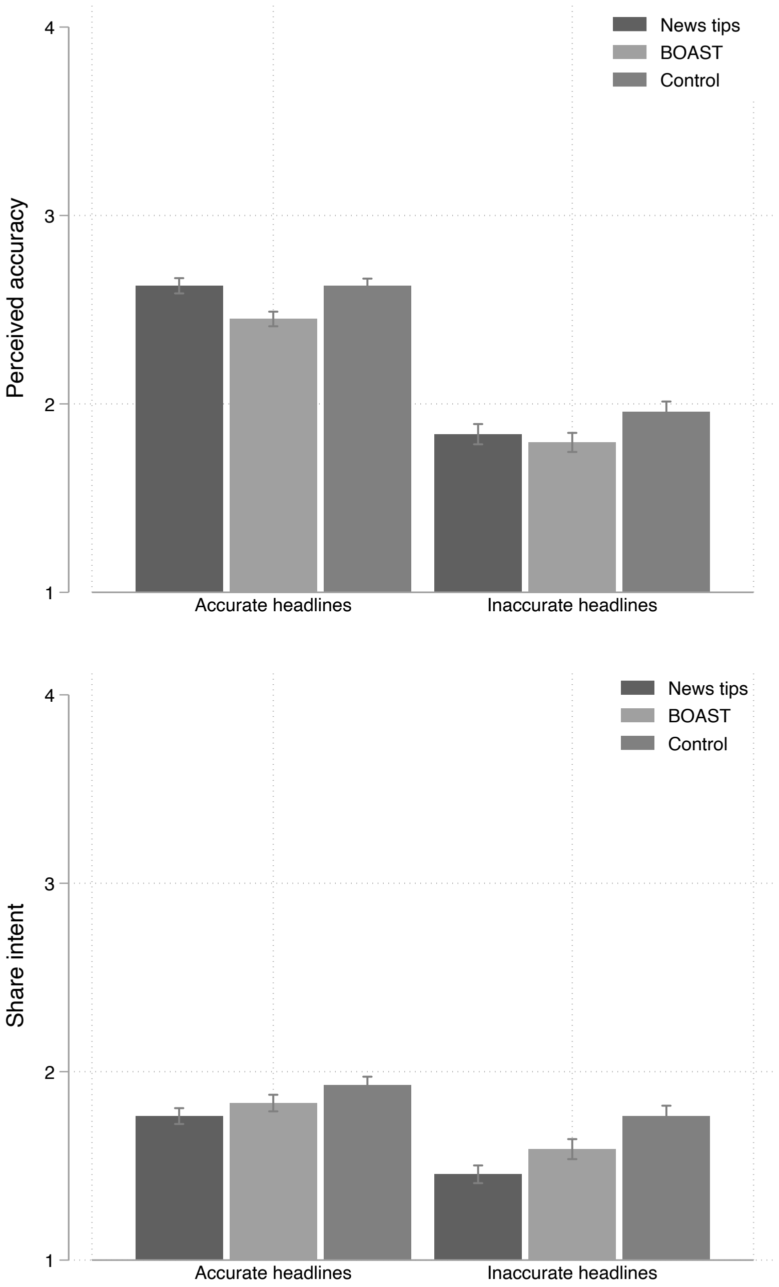

Figure D.2: **Intervention effects on accurate and inaccurate headlines.** Error bars are 95% confidence intervals of the mean.

Table D.6: Intervention effects on sharing intent (binary)

	Inaccurate headlines	Accurate headlines	Difference score
News tips	-0.0981***	-0.0466	0.0559*
	(0.0291)	(0.0328)	(0.0226)
BOAST	-0.0475	-0.0396	0.0153
	(0.0310)	(0.0327)	(0.0216)
Constant	0.2543***	0.4405***	0.0473***
	(0.0319)	(0.0378)	(0.0149)
Headline fixed effects	✓	✓	
BOAST - news tips	0.0506	0.0070	-0.0405
	(0.0275)	(0.0308)	(.0232)
N	3640	6185	607

* $p < .05$, ** $p < .01$, *** $p < .005$ (two-sided). Cell entries are OLS coefficients.

Table D.7: Intervention effects on accuracy and sharing pooled outcome measure

	Inaccurate headlines	Accurate headlines	Difference score
News tips	-0.0812***	-0.0222	0.1340**
	(0.0195)	(0.0141)	(0.0494)
BOAST	-0.0634***	-0.0353**	0.0460
	(0.0192)	(0.0135)	(0.0464)
Constant	0.7416***	1.3583***	0.3951***
	(0.0323)	(0.0427)	(0.0337)
Headline fixed effects	✓	✓	
N	19200	52800	1200

* $p < .05$, ** $p < .01$, *** $p < .005$ (two-sided). Cell entries are OLS coefficients.

Table D.8: Differential intervention effects by outcome measure

	Inaccurate headlines	Accurate headlines	Difference score
News tips	-0.1148***	-0.0408*	0.1457**
	(0.0285)	(0.0202)	(0.0546)
BOAST	-0.0653*	-0.0263	0.0912
	(0.0301)	(0.0204)	(0.0522)
Assigned to accuracy DV	0.0740**	0.1746***	0.5119***
	(0.0280)	(0.0177)	(0.0626)
Tips $\times$ accuracy assignment	0.0701	0.0409	-0.0161
	(0.0380)	(0.0245)	(0.0928)
BOAST $\times$ accuracy assignment	0.0029	-0.0197	-0.0955
	(0.0380)	(0.0243)	(0.0869)
Constant	0.7050***	1.2719***	0.1417***
	(0.0365)	(0.0444)	(0.0366)
Headline fixed effects	✓	✓	
N	19200	52800	1200

* $p < .05$, ** $p < .01$, *** $p < .005$ (two-sided). Cell entries are OLS coefficients.

Table D.9: Differential intervention effects on sharing intent for inaccurate headlines

News tips	0.0257<br>(0.2500)	0.0755<br>(0.6099)	-0.0885<br>(0.2102)	-0.0842<br>(0.1930)	-0.2843***<br>(0.0798)	-0.1471<br>(0.1387)	-0.1436<br>(0.2010)	-0.3135<br>(0.1730)	-0.1385<br>(0.1232)	-0.3156*<br>(0.1250)
BOAST	-0.0725<br>(0.2540)	0.0135<br>(0.5915)	-0.0475<br>(0.2266)	0.0017<br>(0.2075)	-0.1710*<br>(0.0847)	-0.1117<br>(0.1456)	-0.1746<br>(0.2087)	-0.0936<br>(0.1961)	-0.1224<br>(0.1239)	-0.2909*<br>(0.1334)
Low end digital lit.	0.2795**<br>(0.1013)									
Tips × digital lit.	-0.1726<br>(0.1256)									
BOAST × digital lit.	-0.0536<br>(0.1290)									
Health lit.		-0.1939*<br>(0.0977)								
Tips × health lit.		-0.0885<br>(0.1341)								
BOAST × health lit.		-0.0401<br>(0.1286)								
Anti-expert			0.2970***<br>(0.0664)							
Tips × anti-expert			-0.0760<br>(0.0865)							
BOAST × anti-expert			-0.0392<br>(0.0925)							
Conspiracism				0.2279***<br>(0.0515)						
Tips × conspiracy				-0.0657<br>(0.0689)						
BOAST × conspiracy				-0.0473<br>(0.0728)						
Cancer history					0.0759<br>(0.1856)					
Tips × cancer					-0.1973<br>(0.2430)					
BOAST × cancer					-0.0564<br>(0.2597)					
Affect to pharma						0.0053*<br>(0.0024)				
Tips × pharma						-0.0041<br>(0.0031)				
BOAST × pharma						-0.0022<br>(0.0033)				
Affect to doctors							-0.0012<br>(0.0023)			
Tips × doctors							-0.0027<br>(0.0029)			
BOAST × doctors							-0.0000<br>(0.0031)			
Affect to scientists								-0.0017<br>(0.0021)		
Tips × scientist								-0.0000<br>(0.0026)		
BOAST × scientist								-0.0013<br>(0.0028)		
Affect to the media									0.0043*<br>(0.0019)	
Tips × media									-0.0040<br>(0.0025)	
BOAST × media									-0.0012<br>(0.0028)	
Affect to social media										0.0049*<br>(0.0024)
Tips × social media										0.0002<br>(0.0030)
BOAST × social media										0.0029<br>(0.0033)
Constant	1.3144***<br>(0.2041)	2.7032***<br>(0.4427)	1.0612***<br>(0.1767)	1.1374***<br>(0.1581)	1.8490***<br>(0.0802)	1.6505***<br>(0.1201)	1.9292***<br>(0.1605)	1.9565***<br>(0.1482)	1.6769***<br>(0.1042)	1.6768***<br>(0.1112)
Headline fixed effects	✓	✓	✓	✓	✓	✓	✓	✓	✓	✓
N	3640	3640	3640	3640	3640	3520	3640	3640	3640	3640

* $p < .05$, ** $p < .01$, *** $p < .005$ (two-sided). Cell entries are OLS coefficients.

Table D.10: Differential intervention effects on sharing intent for accurate headlines

News tips	0.1002	0.2704	0.0970	-0.0247	-0.1177	0.0394	0.0973	-0.0683	0.0045	-0.2591*
	(0.2546)	(0.6140)	(0.2638)	(0.2500)	(0.0848)	(0.1400)	(0.2141)	(0.1892)	(0.1258)	(0.1315)
BOAST	-0.0575	0.4703	0.0239	-0.0427	-0.0578	0.0408	-0.0237	-0.2680	-0.0086	-0.2818*
	(0.2582)	(0.5885)	(0.2691)	(0.2427)	(0.0861)	(0.1493)	(0.2061)	(0.1842)	(0.1256)	(0.1361)
Low-end digital lit.	0.2199*									
	(0.0959)									
Tips × digital lit.	-0.1367									
	(0.1229)									
BOAST × digital lit.	-0.0207									
	(0.1252)									
Health lit.		-0.1513								
		(0.0980)								
Tips × health lit.		-0.0999								
		(0.1366)								
BOAST × health lit.		-0.1252								
		(0.1298)								
Anti-expert			0.1130							
			(0.0740)							
Tips × anti-expert			-0.0983							
			(0.1000)							
BOAST × anti-expert			-0.0433							
			(0.1037)							
Conspiracism				0.0633						
				(0.0556)						
Tips × conspiracy				-0.0441						
				(0.0791)						
BOAST × conspiracy				-0.0151						
				(0.0788)						
Cancer					0.2265					
					(0.1895)					
Tips × cancer					-0.3834					
					(0.2608)					
BOAST × cancer					-0.3905					
					(0.2704)					
Affect to pharma						0.0078***				
						(0.0023)				
Tips × pharma						-0.0049				
						(0.0031)				
BOAST × pharma						-0.0038				
						(0.0033)				
Affect to doctors							0.0012			
							(0.0024)			
Tips × doctors							-0.0043			
							(0.0033)			
BOAST × doctors							-0.0012			
							(0.0032)			
Affect to scientists								0.0007		
								(0.0022)		
Tips × scientists								-0.0017		
								(0.0029)		
BOAST × scientists								0.0027		
								(0.0028)		
Affect to media									0.0075***	
									(0.0019)	
Tips × media									-0.0039	
									(0.0026)	
BOAST × media									-0.0019	
									(0.0028)	
Affect to social media										0.0046*
										(0.0023)
Tips × social media										0.0024
										(0.0031)
BOAST × social media										0.0047
										(0.0032)
Constant	1.8934***	2.9762***	2.0310***	2.1340***	2.3083***	2.0144***	2.2635***	2.2936***	1.9780***	2.1066***
	(0.2084)	(0.4385)	(0.2082)	(0.1851)	(0.0903)	(0.1238)	(0.1682)	(0.1545)	(0.1126)	(0.1203)
Headline fixed effects	✓	✓	✓	✓	✓	✓	✓	✓	✓	✓
N	6185	6185	6185	6185	6185	5980	6185	6185	6185	6185

* $p < .05$, ** $p < .01$, *** $p < .005$ (two-sided). Cell entries are OLS coefficients.

Table D.11: Differential intervention effects on sharing discernment

News tips	0.0893	0.1325	0.1804	0.0497	0.1681***	0.1909	0.2039	0.2401	0.1356	0.0501
	(0.1587)	(0.3277)	(0.1898)	(0.1903)	(0.0595)	(0.1016)	(0.1622)	(0.1534)	(0.0800)	(0.0913)
BOAST	0.0463	0.4276	0.0514	-0.0514	0.1206*	0.1915*	0.1404	-0.1577	0.1025	0.0151
	(0.1511)	(0.3142)	(0.1667)	(0.1650)	(0.0553)	(0.0967)	(0.1606)	(0.1429)	(0.0897)	(0.0865)
Low-end digital lit.	-0.0575									
	(0.0511)									
Tips × digital lit.	0.0292									
	(0.0739)									
BOAST × digital lit.	0.0228									
	(0.0726)									
Health lit.		0.0370								
		(0.0460)								
Tips × health lit.		0.0031								
		(0.0730)								
BOAST × health lit.		-0.0757								
		(0.0702)								
Anti-expert			-0.1852***							
			(0.0400)							
Tips × anti-expert			-0.0198							
			(0.0660)							
BOAST × anti-expert			0.0081							
			(0.0572)							
Conspiracism				-0.1682***						
				(0.0352)						
Tips × conspiracy				0.0250						
				(0.0562)						
BOAST × conspiracy				0.0382						
				(0.0487)						
Cancer					0.1308					
					(0.0943)					
Tips × cancer					-0.1810					
					(0.1457)					
BOAST × cancer					-0.2942					
					(0.1743)					
Affect to pharma						0.0026				
						(0.0014)				
Tips × pharma						-0.0010				
						(0.0022)				
BOAST × pharma						-0.0024				
						(0.0020)				
Affect to doctors							0.0017			
							(0.0017)			
Tips × doctors							-0.0010			
							(0.0024)			
BOAST × doctors							-0.0008			
							(0.0024)			
Affect to scientists								0.0024		
								(0.0017)		
Tips × scientists								-0.0015		
								(0.0023)		
BOAST × scientists								0.0038		
								(0.0022)		
Affect to media									0.0028*	
									(0.0013)	
Tips × media									0.0002	
									(0.0019)	
BOAST × media									-0.0002	
									(0.0019)	
Affect to social media										-0.0007
										(0.0013)
Tips × social media										0.0025
										(0.0020)
BOAST × social media										0.0020
										(0.0019)
Constant	0.2549*	-0.0216	0.6352***	0.6717***	0.1262***	0.0412	0.0383	0.0019	0.0194	0.1673**
	(0.1071)	(0.2053)	(0.1228)	(0.1245)	(0.0399)	(0.0702)	(0.1131)	(0.1115)	(0.0577)	(0.0638)
N	607	607	607	607	607	587	607	607	607	607

* $p < .05$, ** $p < .01$, *** $p < .005$ (two-sided). Cell entries are OLS coefficients.

Table D.12: Differential intervention effects on perceived accuracy of inaccurate headlines

News tips	0.1377	-0.2956	-0.2820	-0.2457	-0.1220	-0.1453	-0.2172	-0.2094	-0.0888	-0.2907*
	(0.2118)	(0.4671)	(0.1709)	(0.1561)	(0.0718)	(0.1286)	(0.1871)	(0.1788)	(0.1189)	(0.1168)
BOAST	0.1311	0.1851	-0.2032	0.0762	-0.1804**	-0.2106	-0.2148	-0.2993	-0.1129	-0.2923**
	(0.1929)	(0.4019)	(0.1625)	(0.1666)	(0.0668)	(0.1207)	(0.1715)	(0.1610)	(0.1127)	(0.1101)
Low-end digital lit.	0.2403***									
	(0.0723)									
Tips × digital lit.	-0.1342									
	(0.1047)									
BOAST × digital lit.	-0.1495									
	(0.0931)									
Health lit.		-0.1486*								
		(0.0685)								
Tips × health lit.		0.0385								
		(0.1041)								
BOAST × health lit.		-0.0794								
		(0.0907)								
Anti-expert			0.2781***							
			(0.0419)							
Tips × anti-expert			0.0451							
			(0.0668)							
BOAST × anti-expert			0.0160							
			(0.0633)							
Conspiracism				0.3023***						
				(0.0369)						
Tips × conspiracy				0.0289						
				(0.0548)						
BOAST × conspiracy				-0.0887						
				(0.0549)						
cancer					-0.1117					
					(0.1279)					
Tips × cancer					-0.1327					
					(0.1717)					
BOAST × cancer					0.0977					
					(0.1704)					
Affect to pharma						-0.0003				
						(0.0020)				
Tips × pharma						-0.0003				
						(0.0027)				
BOAST × pharma						0.0008				
						(0.0026)				
Affect to doctors							-0.0044*			
							(0.0018)			
Tips × doctors							0.0014			
							(0.0028)			
BOAST × doctors							0.0009			
							(0.0025)			
Affect to scientists								-0.0063***		
								(0.0018)		
Tips × scientists								0.0016		
								(0.0026)		
BOAST × scientists								0.0023		
								(0.0024)		
Affect to media									-0.0011	
									(0.0017)	
Tips × media									-0.0010	
									(0.0025)	
BOAST × media									-0.0013	
									(0.0023)	
Affect to social media										0.0013
										(0.0018)
Tips × social media										0.0041
										(0.0027)
BOAST × social media										0.0029
										(0.0024)
Constant	1.7125***	2.8408***	1.4521***	1.3109***	2.2187***	2.2230***	2.4627***	2.5673***	2.2478***	2.1544***
	(0.1551)	(0.3097)	(0.1238)	(0.1228)	(0.0733)	(0.1064)	(0.1380)	(0.1327)	(0.1001)	(0.0978)
Headline fixed effects	✓	✓	✓	✓	✓	✓	✓	✓	✓	
N	3556	3556	3556	3556	3556	3400	3556	3556	3556	3556

* $p < .05$, ** $p < .01$, *** $p < .005$ (two-sided). Cell entries are OLS coefficients.

Table D.13: Differential intervention effects on perceived accuracy of accurate headlines

News tips	-0.1161	-0.0049	0.1185	0.0398	0.0059	0.0140	0.0110	-0.0063	-0.0082	0.0171
	(0.2776)	(0.3649)	(0.1768)	(0.1786)	(0.0546)	(0.0932)	(0.1412)	(0.1398)	(0.0971)	(0.0873)
BOAST	-0.0539	0.0997	-0.0553	-0.0783	-0.1804***	-0.1813	-0.2265	-0.1995	-0.3103***	-0.1964*
	(0.2510)	(0.3369)	(0.1666)	(0.1677)	(0.0538)	(0.1007)	(0.1545)	(0.1318)	(0.0921)	(0.0956)
Low-end digital lit.	0.0686*									
	(0.0330)									
Tips × health lit.	0.0280	0.0028								
	(0.0611)	(0.0817)								
BOAST × health lit.	-0.0249	-0.0600								
	(0.0561)	(0.0763)								
Health lit.		0.0073								
		(0.0551)								
Anti-expert			-0.0727							
			(0.0394)							
Tips × anti-expert			-0.0379							
			(0.0648)							
BOAST × anti-expert			-0.0428							
			(0.0645)							
Conspiracism				-0.0624						
				(0.0343)						
Tips × conspiracy				-0.0087						
				(0.0564)						
BOAST × conspiracy				-0.0262						
				(0.0541)						
Cancer					-0.0406					
					(0.1039)					
Tips × cancer					0.0019					
					(0.1669)					
BOAST × cancer					0.1175					
					(0.1483)					
Affect to pharma						0.0004				
						(0.0014)				
Tips × pharma						-0.0000				
						(0.0020)				
BOAST × pharma						0.0006				
						(0.0021)				
Affect to doctors							0.0012			
							(0.0013)			
Tips × doctors							-0.0000			
							(0.0021)			
BOAST × doctors							0.0009			
							(0.0022)			
Affect to scientists								0.0003		
								(0.0013)		
Tips × scientists								0.0002		
								(0.0021)		
BOAST × scientists								0.0006		
								(0.0019)		
Affect to media									0.0015	
									(0.0014)	
Tips × media									0.0004	
									(0.0020)	
BOAST × media									0.0034	
									(0.0019)	
Affect to social media										0.0019
										(0.0014)
Tips × social media										-0.0003
										(0.0020)
BOAST × social media										0.0006
										(0.0020)
Constant	2.5534***	2.6600***	2.8810***	2.8783***	2.6974***	2.6724***	2.6216***	2.6722***	2.6290***	2.6217***
	(0.0840)	(0.2483)	(0.1131)	(0.1160)	(0.0537)	(0.0714)	(0.0987)	(0.0990)	(0.0779)	(0.0724)
Headline fixed effects	✓	✓	✓	✓	✓	✓	✓	✓	✓	
N	6180	6180	6180	6180	6180	5910	6180	6180	6180	6180

* $p < .05$, ** $p < .01$, *** $p < .005$ (two-sided). Cell entries are OLS coefficients.

Table D.14: Differential intervention effects on accuracy discernment

News tips	-0.2108	0.0994	0.3845	0.2409	0.1298	0.1819	0.2488	0.1785	0.0824	0.2858*
	(0.2348)	(0.3879)	(0.2067)	(0.2117)	(0.0817)	(0.1453)	(0.2121)	(0.2017)	(0.1348)	(0.1325)
BOAST	-0.4289*	-0.0607	0.1080	-0.2040	-0.0077	0.0321	0.0091	0.1114	-0.2000	0.0619
	(0.2144)	(0.3478)	(0.1772)	(0.2020)	(0.0768)	(0.1424)	(0.2156)	(0.2041)	(0.1306)	(0.1340)
Low-end digital lit.	-0.2103***									
	(0.0741)									
Tips × digital lit.	0.1710									
	(0.1076)									
BOAST × digital lit.	0.2132*									
	(0.0963)									
Health lit.		0.1451*								
		(0.0585)								
Tips × health lit.		0.0060								
		(0.0910)								
BOAST × health lit.		0.0122								
		(0.0812)								
Anti-expert			-0.3618***							
			(0.0485)							
Tips × anti-expert			-0.0794							
			(0.0733)							
BOAST × anti-expert			-0.0465							
			(0.0635)							
Conspiracism				-0.3753***						
				(0.0437)						
Tips × conspiracy				-0.0256						
				(0.0661)						
BOAST × conspiracy				0.0766						
				(0.0634)						
Cancer					0.1152					
					(0.1125)					
Tips × cancer					0.0523					
					(0.2032)					
BOAST × cancer					0.0262					
					(0.1707)					
Affect to pharma						0.0010				
						(0.0019)				
Tips × pharma						-0.0003				
						(0.0029)				
BOAST × pharma						-0.0005				
						(0.0027)				
Affect to doctors							0.0058**			
							(0.0022)			
Tips × doctors							-0.0019			
							(0.0031)			
BOAST × doctors							-0.0004			
							(0.0031)			
Affect to scientists								0.0069***		
								(0.0021)		
Tips × scientists								-0.0012		
								(0.0030)		
BOAST × scientists								-0.0020		
								(0.0030)		
Affect to media									0.0030	
									(0.0018)	
Tips × media									0.0011	
									(0.0027)	
BOAST × media									0.0047	
									(0.0026)	
Affect to social media										-0.0002
										(0.0019)
Tips × social media										-0.0040
										(0.0027)
BOAST × social media										-0.0016
										(0.0026)
Constant	1.0723***	0.0163	1.6008***	1.7677***	0.6379***	0.6102***	0.3018	0.2449	0.5266***	0.6609***
	(0.1624)	(0.2447)	(0.1322)	(0.1347)	(0.0569)	(0.1044)	(0.1567)	(0.1447)	(0.0992)	(0.0950)
N	593	593	593	593	593	567	593	593	593	593

* $p < .05$, ** $p < .01$, *** $p < .005$ (two-sided). Cell entries are OLS coefficients.

Table D.15: Intervention effects on sharing intent by cancer beliefs

	Inaccurate	Accruate	Sharing discernment	Inaccurate	Accurate	Sharing discernment
News tips	-0.4250	-0.4263	0.0618	-0.0414	-0.7872***	-0.7083***
	(0.3840)	(0.4195)	(0.2650)	(0.2018)	(0.2691)	(0.2095)
BOAST	0.0878	0.3097	0.2029	0.5345*	-0.0173	-0.5928***
	(0.4261)	(0.4342)	(0.2556)	(0.2688)	(0.2941)	(0.1915)
Cancer beliefs (true)	0.0292	0.1951*	0.1719***			
	(0.0939)	(0.0976)	(0.0529)			
BOAST × Cancer beliefs (true)	-0.0791	-0.1245	-0.0365			
	(0.1262)	(0.1291)	(0.0779)			
Tips × Cancer beliefs (true)	0.0344	0.0768	0.0244			
	(0.1167)	(0.1271)	(0.0822)			
Cancer beliefs (false)				0.4475***	0.2608***	-0.1846***
				(0.0455)	(0.0501)	(0.0348)
BOAST × Cancer beliefs (false)				-0.2028**	-0.0195	0.1986***
				(0.0753)	(0.0840)	(0.0558)
Tips × Cancer beliefs (false)				-0.0826	0.1849*	0.2568***
				(0.0594)	(0.0811)	(0.0628)
Constant	1.7636***	1.6797***	-0.4294*	0.6547***	1.6150***	0.6395***
	(0.3140)	(0.3328)	(0.1698)	(0.1323)	(0.1570)	(0.1046)
Headline fixed effects	✓	✓		✓	✓	
N	3640	6185	607	3640	6185	607

* $p < .05$, ** $p < .01$, *** $p < .005$ (two-sided). Cell entries are OLS coefficients.

Table D.16: Intervention effects on accuracy perceptions by cancer beliefs

	Inaccurate	Accurate	Accuracy discernment	Inaccurate	Accurate	Accuracy discernment
News tips	-0.6464	-0.1777	0.4712	0.4960*	-0.7892***	-1.2505***
	(0.3304)	(0.2509)	(0.3235)	(0.2298)	(0.1954)	(0.2397)
BOAST	-0.4891	0.0684	0.4802	0.6087*	-0.5419*	-1.2009***
	(0.3245)	(0.2707)	(0.3710)	(0.2417)	(0.2124)	(0.2995)
Cancer beliefs (true)	-0.2064***	0.1870***	0.3835***			
	(0.0628)	(0.0480)	(0.0641)			
BOAST × Cancer beliefs (true)	0.1037	-0.0764	-0.1589			
	(0.0944)	(0.0801)	(0.1096)			
Tips × Cancer beliefs (true)	0.1601	0.0553	-0.1077			
	(0.0994)	(0.0734)	(0.1004)			
Cancer beliefs (false)				0.3820***	-0.0045	-0.3868***
				(0.0361)	(0.0324)	(0.0390)
BOAST × Cancer beliefs (false)				-0.2454***	0.1122	0.3704***
				(0.0686)	(0.0629)	(0.0849)
Tips × Cancer beliefs (false)				-0.2091***	0.2444***	0.4409***
				(0.0667)	(0.0576)	(0.0715)
Constant	2.8756***	2.0808***	-0.5867**	1.2155***	2.6990***	1.6464***
	(0.2213)	(0.1733)	(0.2100)	(0.1117)	(0.1003)	(0.1149)
Headline fixed effects	✓	✓		✓	✓	
N	3556	6180	593	3556	6180	593

* $p < .05$, ** $p < .01$, *** $p < .005$ (two-sided). Cell entries are OLS coefficients.

Table D.17: Correlates of cancer risk belief

	True risks	False risks	Difference score
Age 30-44	-0.0402	0.1273	-0.1675*
	(0.0721)	(0.0723)	(0.0770)
Age 45-59	-0.2128***	-0.0138	-0.1990*
	(0.0727)	(0.0729)	(0.0777)
Age 60+	-0.2390***	-0.0251	-0.2139**
	(0.0735)	(0.0737)	(0.0785)
Female	0.0300	0.1189***	-0.0890*
	(0.0395)	(0.0395)	(0.0421)
College	0.2219***	0.0908*	0.1311***
	(0.0398)	(0.0399)	(0.0425)
Nonwhite	-0.0989*	0.1088*	-0.2077***
	(0.0443)	(0.0444)	(0.0473)
Cancer history	0.0053	-0.0772	0.0825
	(0.0615)	(0.0616)	(0.0656)
"Low end" digital literacy	0.0201	0.0347	-0.0146
	(0.0305)	(0.0306)	(0.0326)
Health literacy	-0.0233	-0.1502***	0.1270***
	(0.0307)	(0.0307)	(0.0328)
Anti-expert sentiment	-0.1317***	0.1173***	-0.2490***
	(0.0270)	(0.0270)	(0.0288)
Conspiracism	0.0219	0.1889***	-0.1670***
	(0.0237)	(0.0237)	(0.0253)
Affect toward pharma.	-0.0034***	-0.0019*	-0.0014
	(0.0008)	(0.0008)	(0.0008)
Affect toward doctors	0.0025***	0.0001	0.0024**
	(0.0008)	(0.0008)	(0.0009)
Affect toward scientists	0.0005	-0.0004	0.0009
	(0.0008)	(0.0008)	(0.0008)
Affect toward the media	0.0006	0.0001	0.0005
	(0.0008)	(0.0008)	(0.0008)
Affect toward social media	0.0000	0.0025***	-0.0025***
	(0.0008)	(0.0008)	(0.0009)
Constant	3.6698***	2.2278***	1.4420***
	(0.1955)	(0.1959)	(0.2088)
R^2	0.12	0.21	0.30
N	1154	1154	1154

* $p < .05$, ** $p < .01$, *** $p < .005$ (two-sided). Cell entries are OLS coefficients.

Table D.18: Correlates of cancer risk belief (additional predictors)

	True risks	False risks	Difference score
Age 30-44	-0.0265	0.1634*	-0.1899*
	(0.0752)	(0.0758)	(0.0805)
Age 45-59	-0.1654*	0.0335	-0.1989*
	(0.0759)	(0.0766)	(0.0813)
Age 60+	-0.1838*	0.0319	-0.2157**
	(0.0777)	(0.0784)	(0.0832)
Female	0.0393	0.1136**	-0.0744
	(0.0404)	(0.0408)	(0.0433)
College	0.1939***	0.0901*	0.1038*
	(0.0434)	(0.0438)	(0.0464)
Income (16 pt. scale)	0.0123*	-0.0048	0.0171**
	(0.0060)	(0.0060)	(0.0064)
Black	-0.2781***	-0.2195*	-0.0586
	(0.0923)	(0.0931)	(0.0988)
Hispanic	-0.0495	-0.1041	0.0546
	(0.0964)	(0.0972)	(0.1032)
Other nonwhite racial background	0.0061	0.2184***	-0.2123**
	(0.0744)	(0.0751)	(0.0797)
Cancer history	-0.0325	-0.0793	0.0468
	(0.0630)	(0.0635)	(0.0674)
Health insurance	0.0230	0.0087	0.0144
	(0.0718)	(0.0724)	(0.0769)
Social media use (9 pt. scale)	-0.0164	-0.0233*	0.0068
	(0.0098)	(0.0099)	(0.0105)
“Low end” digital literacy	0.0095	0.0345	-0.0249
	(0.0311)	(0.0314)	(0.0333)
Health literacy	-0.0349	-0.1580***	0.1231***
	(0.0313)	(0.0316)	(0.0335)
Anti-expert sentiment	-0.1247***	0.1221***	-0.2468***
	(0.0274)	(0.0277)	(0.0294)
Conspiracism	0.0270	0.1832***	-0.1562***
	(0.0243)	(0.0245)	(0.0260)
Affect toward pharma.	-0.0031***	-0.0018*	-0.0013
	(0.0008)	(0.0008)	(0.0008)
Affect toward doctors	0.0023**	0.0004	0.0019*
	(0.0008)	(0.0009)	(0.0009)
Affect toward scientists	0.0009	0.0000	0.0009
	(0.0008)	(0.0008)	(0.0009)
Affect toward the media	0.0012	0.0005	0.0007
	(0.0008)	(0.0008)	(0.0009)
Affect toward social media	0.0002	0.0024***	-0.0022*
	(0.0008)	(0.0008)	(0.0009)
Constant	3.5881***	2.2676***	1.3205***
	(0.2126)	(0.2144)	(0.2276)
R^2	0.14	0.22	0.30
N	1102	1102	1102

* $p < .05$, ** $p < .01$, *** $p < .005$ (two-sided). Cell entries are OLS coefficients.

Table D.19: Intervention effects on perceived accuracy (with controls)

	Inaccurate headlines	Accurate headlines	Difference score
News tips	-0.1855***	0.0068	0.1846***
	(0.0566)	(0.0498)	(0.0627)
BOAST	-0.1867***	-0.1851***	-0.0061
	(0.0526)	(0.0485)	(0.0585)
Cancer beliefs (true)	-0.2858***	0.1904***	0.4716***
	(0.0349)	(0.0350)	(0.0422)
Cancer beliefs (false)	0.4269***	-0.0279	-0.4535***
	(0.0344)	(0.0339)	(0.0356)
Constant	2.0280***	2.1420***	0.2920*
	(0.1497)	(0.1349)	(0.1426)
Headline fixed effects	✓	✓	
N	3556	6180	593

* $p < .05$, ** $p < .01$, *** $p < .005$ (two-sided). Cell entries are OLS coefficients with robust standard errors in parentheses.

Table D.20: Intervention effects on sharing intent (with controls)

	Inaccurate headlines	Accurate headlines	Difference score
News tips	-0.3137***	-0.1700*	0.1447**
	(0.0700)	(0.0777)	(0.0518)
BOAST	-0.1405	-0.0893	0.0617
	(0.0737)	(0.0798)	(0.0495)
Cancer beliefs (true)	-0.1435***	0.0889	0.2367***
	(0.0431)	(0.0520)	(0.0357)
Cancer beliefs (false)	0.4602***	0.2478***	-0.2109***
	(0.0458)	(0.0509)	(0.0350)
Constant	1.0914***	1.3528***	-0.0761
	(0.1754)	(0.2043)	(0.1122)
Headline fixed effects	✓	✓	
N	3640	6185	607

* $p < .05$, ** $p < .01$, *** $p < .005$ (two-sided). Cell entries are OLS coefficients with robust standard errors in parentheses.

E Populated pre-analysis plan

Here we report a populated pre-analysis plan with the location of all pre-registered tests. Note that the hypothesis/RQ wording and ordering below is verbatim from our pre-registration, and varies slightly in the final manuscript text.

Correlations

RQ0: Do digital literacy or health literacy correlate with false cancer news belief, sharing, or discernment? What are other correlates of these outcomes?

- Reported in Table 2, Table D.3, Table D.4 and Figure 3.

Intervention effects

H1. News tips a) reduce belief/sharing in false headlines and b) improve discernment.
H2. BOAST reduces belief/sharing in false headlines and improves discernment.
RQ1. Is the effect of BOAST on belief/sharing or discernment larger than that of new tips?
RQ2. What are the effects of interventions on belief/sharing for true headlines?

- H1, H2, RQ1 and RQ2 tests are jointly reported in Table 3, Table 4, and Figure D.2. Binary outcomes are also reported in Table D.5, Table D.6, and Figure 4.

RQ3. Are the interventions more effective on beliefs than sharing intent?

- Reported in Table D.8.

RQ4. What are the effects of the interventions on news behavior (trace data)?

- To be reported in a separate manuscript.

RQ5. Are the effects of interventions moderated by [conspiracy ideation, anti-expert views, digital literacy, health literacy, cancer history, trust measures, etc]?

- Reported in Tables D.9 through Tables D.16.
